# Microecological Koch’s postulates reveal that intestinal microbiota dysbiosis contributes to shrimp white feces syndrome

**DOI:** 10.1186/s40168-020-00802-3

**Published:** 2020-03-10

**Authors:** Zhijian Huang, Shenzheng Zeng, Jinbo Xiong, Dongwei Hou, Renjun Zhou, Chengguang Xing, Dongdong Wei, Xisha Deng, Lingfei Yu, Hao Wang, Zhixuan Deng, Shaoping Weng, Satapornvanit Kriengkrai, Daliang Ning, Jizhong Zhou, Jianguo He

**Affiliations:** 1 School of Marine Sciences, Sun Yat-sen University, Guangzhou, Guangdong People’s Republic of China; 2 State Key Laboratory of Biocontrol, School of Life Sciences, Sun Yat-sen University, Guangzhou, Guangdong People’s Republic of China; 3 Southern Marine Sciences and Engineering Guangdong Laboratory (Zhuhai), Sun Yat-sen University, Guangzhou, Guangdong People’s Republic of China; 4 South China Sea Resource Exploitation and Protection Collaborative Innovation Center, School of Marine Sciences, Sun Yat-sen University, Guangzhou, Guangdong People’s Republic of China; 5 School of Marine Sciences, Ningbo University, Ningbo, People’s Republic of China; 6 Department of Fishery Biology, Faculty of Fisheries, Kasetsart University, Bangkok, Thailand; 7 Institute for Environmental Genomics, Department of Microbiology and Plant Biology, and School of Civil Engineering and Environmental Sciences, University of Oklahoma, Norman, OK USA

**Keywords:** Pacific white shrimp, Intestinal microbiota, Intestinal microbiota transplantation, White feces syndrome, Microecological Koch’s postulates

## Abstract

**Background:**

Recently, increasing evidence supports that some complex diseases are not attributed to a given pathogen, but dysbiosis in the host intestinal microbiota (IM). The full intestinal ecosystem alterations, rather than a single pathogen, are associated with white feces syndrome (WFS), a globally severe non-infectious shrimp disease, while no experimental evidence to explore the causality. Herein, we conducted comprehensive metagenomic and metabolomic analysis, and intestinal microbiota transplantation (IMT) to investigate the causal relationship between IM dysbiosis and WFS.

**Results:**

Compared to the Control shrimp, we found dramatically decreased microbial richness and diversity in WFS shrimp. Ten genera, such as *Vibrio*, *Candidatus* Bacilloplasma, *Photobacterium*, and *Aeromonas*, were overrepresented in WFS, whereas 11 genera, including *Shewanella*, *Chitinibacter*, and *Rhodobacter* were enriched in control. The divergent changes in these populations might contribute the observation that a decline of pathways conferring lipoic acid metabolism and mineral absorption in WFS. Meanwhile, some sorts of metabolites, especially lipids and organic acids, were found to be related to the IM alteration in WFS. Integrated with multiomics and IMT, we demonstrated that significant alterations in the community composition, functional potentials, and metabolites of IM were closely linked to shrimp WFS. The distinguished metabolites which were attributed to the IM dysbiosis were validated by feed-supplementary challenge. Both homogenous selection and heterogeneous selection process were less pronounced in WFS microbial community assembly. Notably, IMT shrimp from WFS donors eventually developed WFS clinical signs, while the dysbiotic IM can be recharacterized in recipient shrimp.

**Conclusions:**

Collectively, our findings offer solid evidence of the causality between IM dysbiosis and shrimp WFS, which exemplify the ‘microecological Koch’s postulates’ (an intestinal microbiota dysbiosis, a disease) in disease etiology, and inspire our cogitation on etiology from an ecological perspective.

Video abstract

## Background

The traditional ‘Koch’s postulates’ (a pathogen, a disease) have successfully guided pathologists in identifying the causative agents of diverse infectious diseases [1]. Recently, increasing human and animal complex diseases, including non-infectious diseases and some diseases with a syndrome, are not fulfill these concepts, thereby prompting researchers to reconsider that the etiology is multifactorial [2]. Indeed, increasing recognitions on complex diseases (inflammatory bowel disease (IBD), atherosclerotic cardiovascular disease, etc.) illustrate that dysbiosis in intestinal microbiota (IM) contributes to host diseases [3, 4].

IM plays fundamental roles in regulating host metabolic homeostasis, physiology, and health [3, 5, 6]. A few animal models have been used to study the microbe-host crosstalk by fecal microbiota transplantation (FMT) with colonization of specific microbial strains [7]. It is now common knowledge that the intestine is a complex ecosystem with different interacting entities and that infections must be understood in this context rather than isolated as a pathogen and a host. In a very recent review, the ecological Koch’s postulates (a gut ecosystem state, a disease) were proposed [8]. The authors suggest that a whole ecosystem, including host IM, genetic make-up of the host, as well as nutrition and age, etc., forms an entity, ultimately leading to diseases, rather than an isolated microorganism or group of microorganisms [8]. To some extent, for some complex diseases (e.g., aquatic animal diseases) that lack of obvious evidence to be linked to specific mutations or age, but the microbial alterations and the complexity of surrounding environment, neither the Koch’s postulates nor the ecological Koch’s postulates are not sufficiently to interpret. We propose yet another interpretation of Koch’s postulates, which we have termed ‘microecological Koch’s postulates’ (an intestinal microbiota dysbiosis, a disease).

Aquaculture is responsible for the continuing impressive growth in the supply for human consumption, which is the third largest source of animal protein that accounts for 17% protein consumed by the global population [9]. Pacific white shrimp, *Litopenaeus vannamei*, represents the largest production in shrimp industry (global production reached 4.1 million tons and valued at over $24 billion) [10]. However, shrimp production is being threatened by several diseases, such as early mortality syndrome (EMS) [11], acute hepatopancreatic necrosis disease (AHPND) [12], hepatopancreas necrosis syndrome (HPNS) [13], and white feces syndrome (WFS) [14, 15], which cumulatively cause a devastating drop (60%) in shrimp production, of which WFS is the most severe and has drawn wide attention. WFS etiology has been widely concerned. The microsporidian was firstly proposed as causality of WFS [16, 17], while this hypothesis was not supported by subsequent study [18]. Another survey on *Penaeus monodon* demonstrates that several *Vibrio* species may be the major causative pathogen of WFS, and the occurrence of WFS are related to the total *Vibrio* count in shrimp intestine [19]. By comparing the IM between healthy and WFS shrimp, increasing evidences have shown that the IM of WFS shrimp are less diverse and significantly different from those of healthy shrimp, and shrimp WFS is correlated with dysbiosis in IM [19, 20, 21]. Accordingly, WFS is a non-infectious disease that is not attributed by one pathogen, but a bacterial continuum. However, it is still unclear whether IM dysbiosis is a consequence or the causality of WFS. We therefore hypothesized that IM dysbiosis is the causality of WFS.

To address the hypothesis above, in the present study, a comprehensive exploration of the IM aberrations in WFS shrimp was achieved by combining compositional, functional, and metabolic data. The causal link of IM dysbiosis and WFS was firstly validated by reciprocal intestinal microbiota transplantation (IMT) between healthy and WFS shrimp, which preferably fit the concept of ‘microecological Koch’s postulates.’ These valuable findings greatly enhanced our understanding on the etiology in host disease from an ecological perspective.

## Results

### Clinical signs and histopathology of WFS

**Fig. 1:**
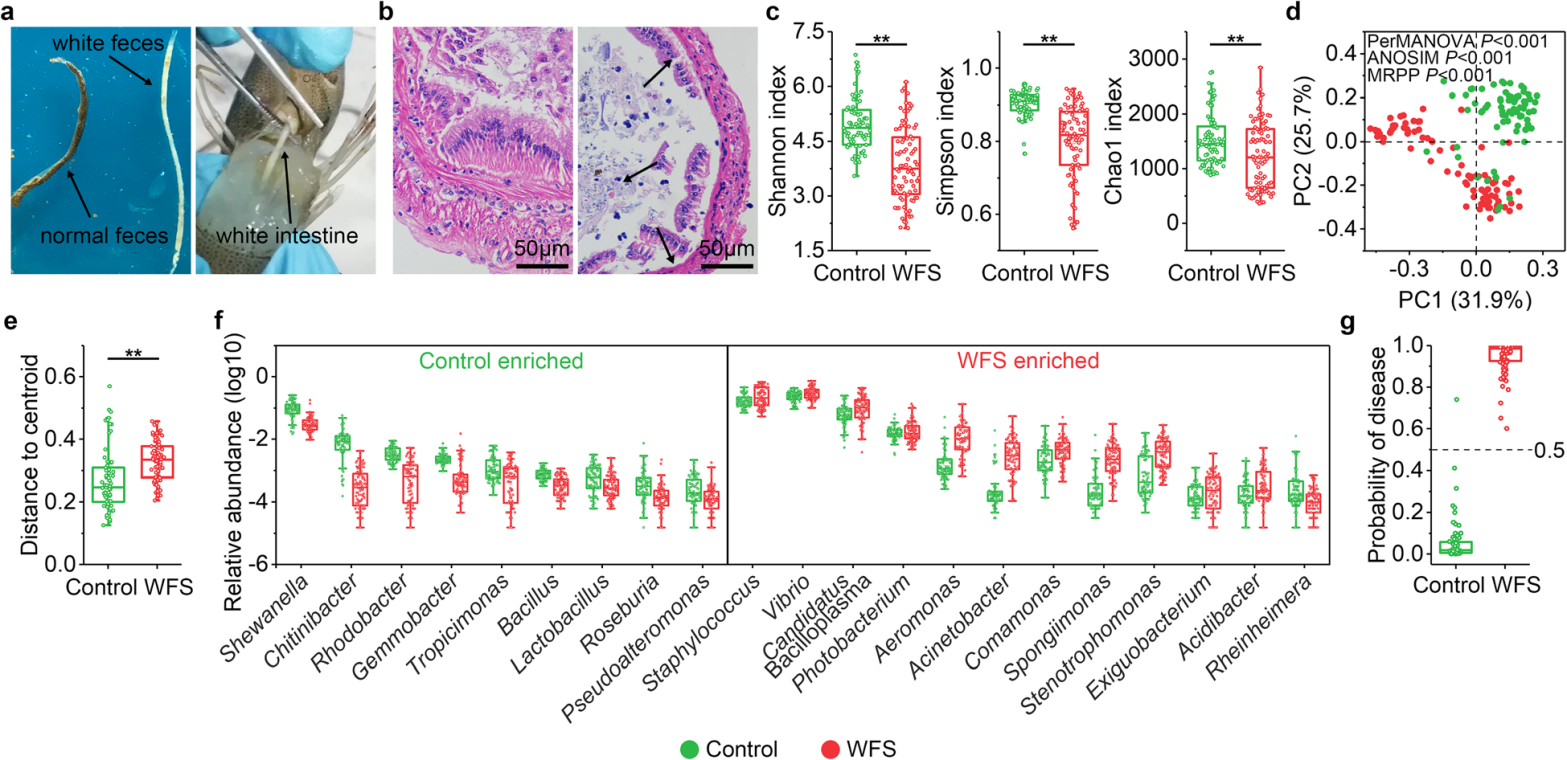
Characterization of the clinical signs, histological pathology, and microbial features of WFS. **a** The diseased shrimp excretes white feces. The intestine of WFS shrimp is distended and filled with white content. **b** Comparison of histological pathology of shrimp intestine with or without WFS signs. The black arrows point towards pathological features, including dropped epithelial cell, reduced or disappeared microvilli, and thinner mid intestine. **c** The *α*-diversity comparison between control group (*n* = 75) and WFS group (*n* = 84). Shannon index, *P* = 0.002; Simpson index, *P* < 0.001; Chao1 index, *P* = 0.002. Significant differences are indicated by asterisks (*, *P* < 0.05; **, *P* < 0.01). **d** Samples were clustered into two groups by PCoA based on Bray-Curtis distance. The microbial structure differed significantly between control group and WFS group confirmed by PerMANOVA (*P* < 0.001), ANOSIM (*P* < 0.001), and MRPP (*P* < 0.001). **e** Comparison of *β*-dispersion based on Bray-Curtis distance between Control and WFS, *P* = 0.008 (Student’s *t*-test). **f** Genera are strikingly different between control group and WFS group. The box plot shows that the relative abundance of ten genera were enriched in Control and the relative abundance of 11 genera were abundant in WFS. **g** The random forests model was conducted to predict the probability of disease (POD). The diagnosed probability of shrimp WFS was based on profiles of the disease-discriminatory taxa. The probability > 50% was stratified as WFS shrimp, while < 50% was stratified as control shrimp

The comprehensive exploration of the IM aberrations in WFS shrimp was performed following the simple workflow (Additional file 2: Figure S1). The symptoms of WFS shrimp include weakened activity, no feed intake, and excreting white feces. White floating fecal strings were found at the water surface in ponds with WFS populations. The mid intestine of WFS shrimp was distended and filled with white contents, whereas that of healthy shrimp was brown and filled with feed (Fig. 1a). Regarding to the histological pathology, the intestine of WFS shrimp contained epithelial cell detachment, reduced or disappeared microvilli, and thinner mid intestine (Fig. 1b).

### Comparison of the IM composition between control and WFS

In total, there were 26,350,076 high-quality reads, with an average of 84,186 ± 5404 reads, which resulting in 12,204 operational taxonomic units (OTUs) across the enrolled 75 Control (no clinical signs) and 84 WFS samples (Additional file 1: Table S1). OTUs were classified into 61 phyla (Additional file 2: Figure S2a) and 1135 genera (Additional file 2: Figure S2b).

The *α*-diversity in WFS was significantly lower than that in control, as supported by Shannon index (*P* < 0.001), Simpson index (*P* < 0.001), and Chao1 index (*P* < 0.001) (Fig. 1c). A principal coordinate analysis (PCoA) revealed that WFS bacterial communities were markedly distinct from control (Fig. 1d). The significant differences in intestinal community structure between control and WFS were confirmed by analysis of similarities (ANOSIM, *P* < 0.001), permutational multivariate analysis of variance (PerMANOVA, *P* < 0.001), and multiple-response permutation procedure (MRPP, *P* < 0.001). Permutational analysis of multivariate dispersions (PERMDISP) showed that significantly higher distances from the PCoA group centroid were displayed in WFS than in control (*P* < 0.01, Fig. 1e), suggesting the IM community structures of WFS are less homogeneous than that of control.

Ten genera, such as *Vibrio*, *Candidatus* Bacilloplasma, *Photobacterium*, and *Aeromonas*, were overrepresented in WFS, whereas 11 genera, including *Shewanella*, *Chitinibacter*, and *Rhodobacter*, were enriched in control (Fig. 1f). Similarity percentage analysis (SIMPER) showed that the *Vibrio*, *Candidatus* Bacilloplasma, *Photobacterium*, *Aeromonas*, *Shewanella*, and *Gemmobacter* contributed 25.9%, 20.3%, 14.0%, 11.0%, 3.9%, and 2.4% dissimilarity of IM between control and WFS, respectively (Additional file 1: Table S2).

A tenfold cross-validation error curve searched 167 top ranking disease-discriminatory OTUs as the minimized numbers of biomarkers based on their feature importance (Additional file 2: Figure S3). The probability of disease index (POD) values based on these markers were significantly higher in WFS than in control (*P* < 0.001), which contributed an overall 99.4% diagnosis accuracy (Fig. 1g).

### Functional alteration in IM of control and WFS

**Fig. 2:**
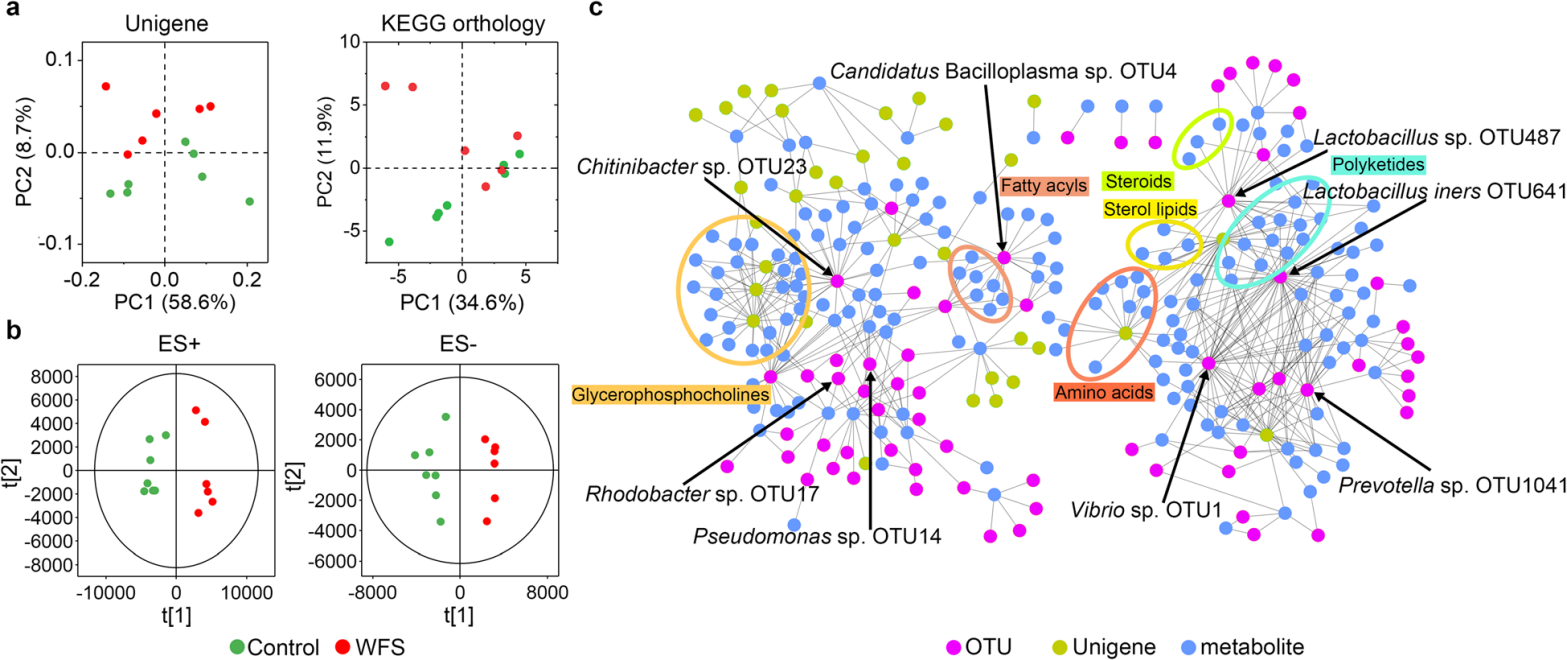
Comparative analysis of microbial gene functions and metabolic patterns between control and WFS. **a** PCoA based on the relative abundance of all Unigenes with Bray-Curtis distance and KEGG orthology groups. **b** OPLS-DA score plots based on the metabolic profiles in intestine samples from control group (*n* = 7) and WFS group (*n* = 6) in ES+ and ES− models. **c** Multiomics data integration for different categories. The relationship (edges) between OTUs (purple), Unigenes (olive) and metabolites (blue) between all samples is estimated by Spearman’s correlation analysis. And those with low correlated (|*r*| < 0.7) are not shown

A total of 68.9 Gb 125-bp paired-end reads were generated from seven control and six WFS representatives, with an average of 5.3 ± 0.83 million reads per sample (Additional file 1: Table S3). Using the Kyoto Encyclopedia of Genes and Genomes (KEGG) database, the IM functions were evaluated across groups (Additional file 1: Table S4). A principal component analysis (PCA) based on genes and KEGG orthology revealed striking differences in microbial functional structures between Control and WFS (Fig. 2a). In total, KEGG orthologs were significantly altered between the two group (*P* < 0.05) (Additional file 2: Figure S4a). Thirteen KEGG pathways, including mineral absorption, aminobenzoate degradation, lipoic acid metabolism, and phenylpropanoid biosynthesis decreased significantly in WFS (*P* < 0.05) compared with control (Additional file 2: Figure S4b). By contrast, 14 pathways, such as bacterial chemotaxis, caprolactam degradation, lysosome, flagellar assembly, and other glycan degradation exhibited the opposing trend.

### Metabolic profiling of IM in control and WFS

To demonstrate the linkages between IM and metabolites, the metabolic profiling was explored using high-throughput liquid chromatography-mass spectrometry (LC/MS). A total of 71 kinds of compounds were listed in KEGG compound database (Additional file 1: Table S5). Orthogonal partial least-squares discriminant analysis (OPLS-DA) revealed distinct metabolic profiles between control and WFS (Fig. 2b). After KEGG annotation, 35 compounds, including cholic acid, lithocholic acid, linoleic acid, myristic acid, sissotrin, daidzein, and glycitein, were significantly different between control and WFS (Additional file 2: Figure S5).

### Integration of microbiome, metagenome, and metabolome

There were significant associations between IM and its mediated metabolites, including nucleic acids (Mantel *r* = 0.242, *P* = 0.033), organic acids (Mantel *r* = 0.263, *P* = 0.021), and lipids (Mantel *r* = 0.287, *P* = 0.015) (Additional file 1: Table S6). Spearman’s correlation analysis depicted the relationship between 35 metabolites and the 21 most different genera (Additional file 2: Figure S6). Ten altered metabolites, for example, including sissotrin, cholic acid, and phenylethylamine, were positively correlated with control-enriched *Chitinibacter*, while nine metabolites (such as capric acid and succinic) were negatively linked to *Chitinibacter*. There were positive associations between ten metabolites (oleic acid, capric acid, succinic, and so on) and WFS-enriched *Vibrio*. Capric acid and succinic were associated with the greatest number of genera that are potential pathogens, such as *Vibrio*, which might be important metabolites involved in the development of WFS. Notably, Procrustes analysis integrating the microbiota, metagenome, and metabolome data showed a strong correspondence between any two data sets (Additional file 2: Figure S7a), accompanied with evenly distributed number of Procrustes residuals between the control and WFS (Additional file 2: Figure S7b).

The intimate association among the IM, metagenome, and metabolome facilitates the identification of specific species, functional genes, and metabolites. The *Vibrio* and *Lactobacillus* were significantly correlated with polyketides, amino acids, and sterol lipids, while *Candidatus* Bacilloplasma and *Chitinibacter* were linked to fatty acyls and glycerophosphocholines (Fig. 2c). The main bacterial hubs (with the greatest number of connections to other nodes) of the entire network were represented by *Vibrio* sp. OTU1, *Candidatus* Bacilloplasma sp. OTU4, *Rhodobacter* sp. OTU17, *Chitinibacter* sp. OTU23, and *Lactobacillus iners* OTU641 (Additional file 1: Table S7), which were also the disease-discriminatory taxa.

### WFS is transferrable by IMT

**Fig. 3:**
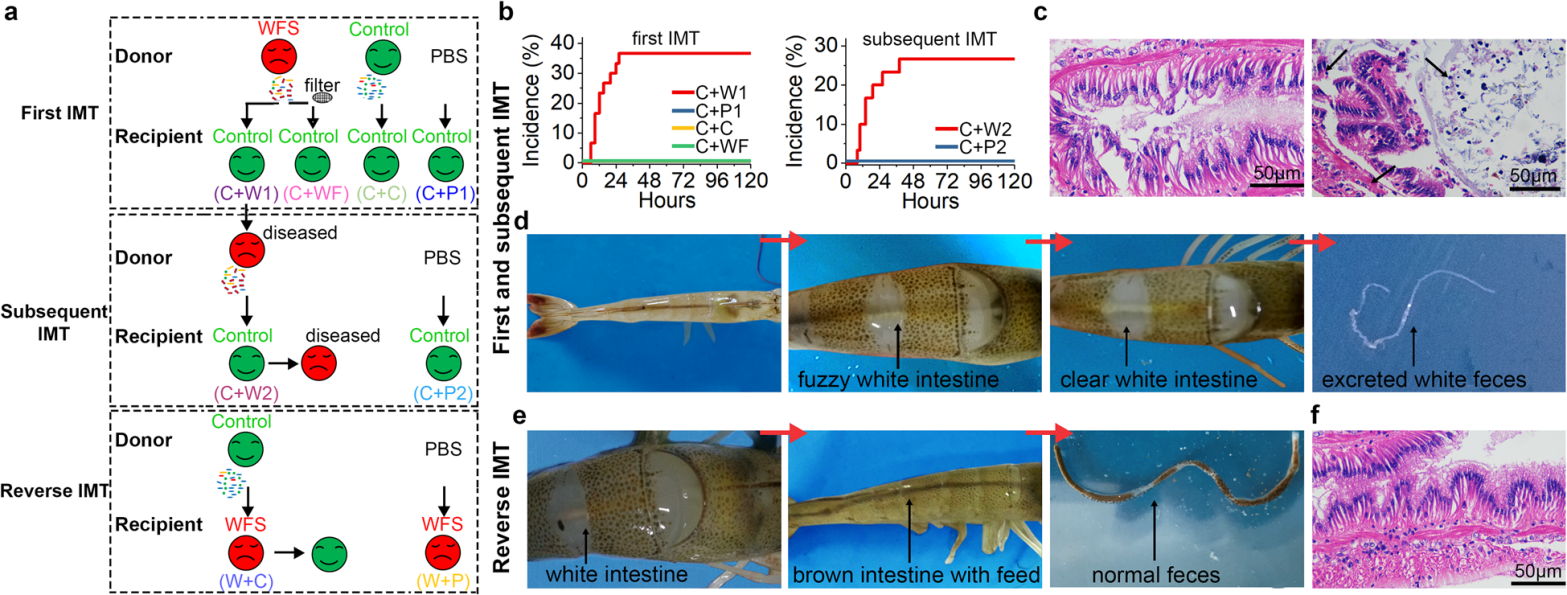
Transplantation of IM leads to similar symptom of WFS. **a** Schematic representation of IMT procedures. Control shrimp (*n* = 30) were received IMT from different donors to evaluate the causative role of IM dysbiosis to WFS. The shrimp suffered from WFS after IMT were selected as WFS donors for the subsequent IMT. To determine whether WFS shrimp can regress by IMT from donors without WFS, WFS shrimp (*n* = 22) were received IMT from Control donors and PBS. **b** Incidence of recipient shrimp suffered from WFS after the first IMT and the subsequent IMT. **c** Histological pathology of WFS shrimp. The black arrows point towards the disease signs of WFS (dropped epithelial cell, reduced, or disappeared microvilli, thinner mid intestine). **d** Observation of the progressive severity of WFS after IMT. **e** The WFS shrimp recovered to health by IMT from control donors. **f** Histological pathology confirmed that the diseased shrimp regressed to health

To further demonstrate whether the aberration of IM is a causal factor in WFS occurrence in vivo, IM from WFS donors (*n* = 6) were transplanted to healthy shrimp (C + W1, *n* = 30) by IMT (Fig. 3a). A total of 11 recipient shrimp (36.7%) developed WFS signs (Fig. 3b, Additional file 1: Table S8). Intestinal histopathology confirmed the typical WFS symptoms in diseased shrimp, including epithelial cell detachment, reduced or disappeared microvilli, and thinner mid intestine (Fig. 3c). However, recipients received phosphate buffer saline (PBS) (C + P1, *n* = 30) or IM of healthy donors (C + C, *n* = 30) did not cause WFS clinical signs over the same timeframe. In addition, control shrimp receiving IM filtrates from WFS donors (C + WF, *n* = 30) were not suffered from WFS, indicating that the virus did not contribute to WFS (Additional file 1: Table S8).

To evaluate whether the dysbiotic IM in the newly WFS shrimp could contribute to WFS, six WFS shrimp in C + W1 group were selected as the donors for the subsequent IMT. Control shrimp were divided into two groups (*n* = 30) to receive IM (C + W2) and PBS (C + P2). Consequently, eight recipients (26.7%) exhibited WFS signs after the second IMT (Fig. 3b, Additional file 1: Table S8), as supported by progressive severity after IMT (Fig. 3d). However, WFS did not occur in C + P2 group. Intriguingly, two groups of WFS shrimp were separately transferred by IMT with control donors (W + C) and PBS (W + P), of which 13 (59.1%) WFS shrimp in W + C group recovered to health (Fig. 3e, f, Additional file 1: Table S8). Collectively, these findings demonstrate that dysbiotic IM causally contributes to WFS.

### Recharacterization of IM dysbiosis in recipient shrimp

**Fig. 4:**
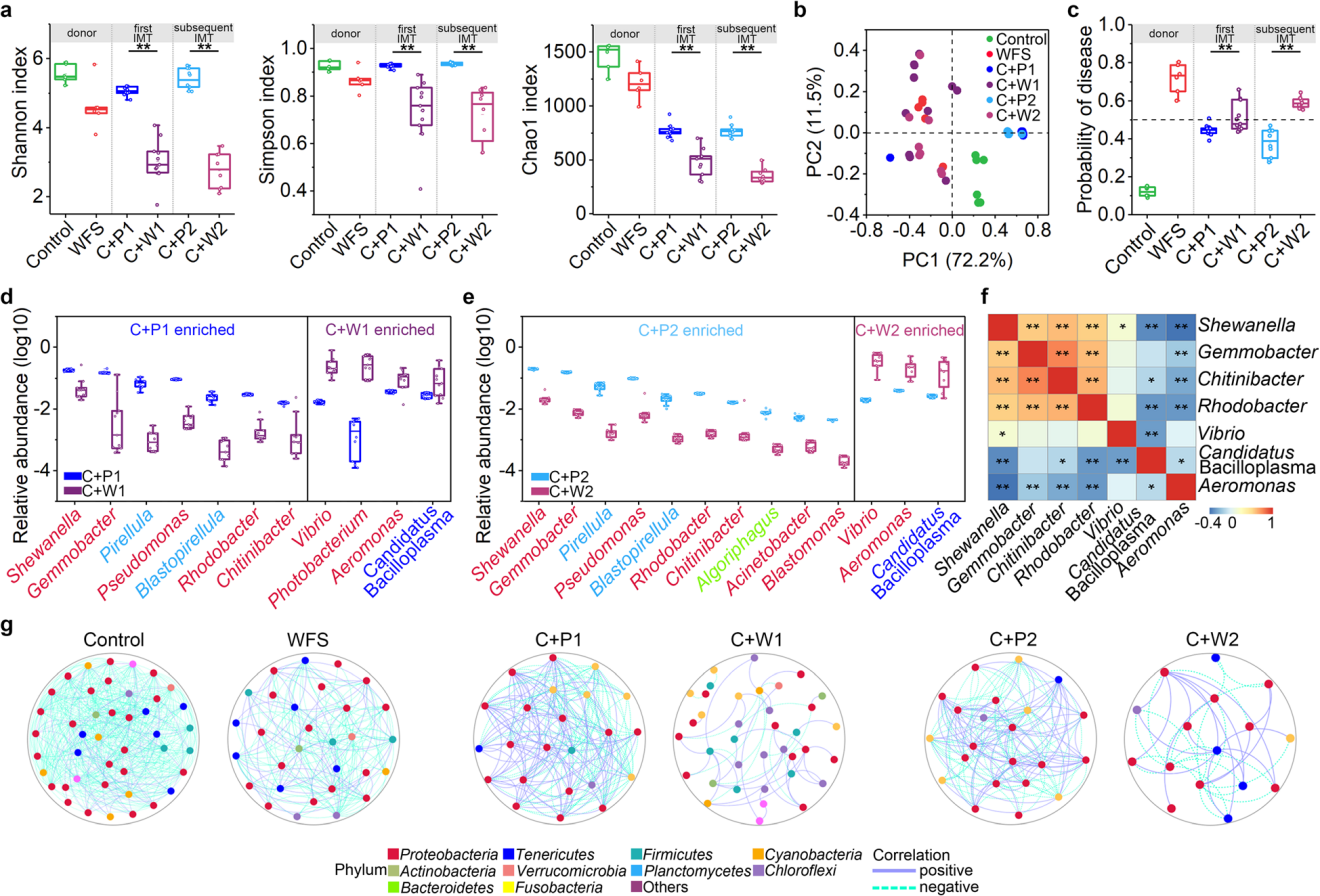
Recharacterize the IM dysbiosis in the newly diseased shrimp. **a** Recipient control shrimp significantly reduced α-diversity (*P* < 0.05, Student’s *t* test). **b** WFS donors and recipient shrimp suffered from WFS were clustered closely, separating from the Control and PBS. **c** The POD value significantly increased after the first and subsequent IMT from WFS donors. **d** Boxplot comparing the abundance of altered genera after receiving the first IMT. **e** Boxplot comparing the abundance of altered genera after receiving the subsequent IMT. **f** Heatmap showed the relationship among the distinguished genera. *P* < 0.05 (Spearman’s correlation). **g** The molecular ecology networks (MENs) of different groups, which showed that the MENs of shrimp suffered from WFS were less species interactions and less complex

The Alpha diversity in C + W1 and C + W2 groups was significantly lower than that in C + P1 and C + P2 ones (Fig. 4a). PCoA biplot revealed that samples from WFS, C + W1, and C + W2 groups closely clustered and were distinct from these of control individuals (Fig. 4b). Again, at bacterial genus level, *Vibrio*, *Photobacterium*, *Candidatus* Bacilloplasma, and *Aeromonas* were confirmed to be abundant in C + W1 and C + W2 groups, while *Shewanella*, *Chitinibacter*, and *Rhodobacter* were more deficient in C + P1 and C + P2 groups (Fig. 4d, e). This pattern was concurrent with the IM composition alterations between healthy and WFS shrimp (Fig. 1). Furthermore, the WFS-enriched *Vibrio*, *Candidatus* Bacilloplasma, and *Aeromonas* were negatively correlated with the control-enriched genera (*Shewanella*, *Gemmobacter*, and *Rhodobacter*) (Spearman’s correlation: *P* < 0.05, Fig. 4f). In the reverse IMT, results showed that the IM of recuperating health shrimp are with higher α-diversity (Additional file 2: Figure S8a) and similar microbial composition to control shrimp (Additional file 2: Figure S8b and c). To validate the diagnostic potential, the POD values for each IMT-treated shrimp were further calculated. Results showed that the POD values were significantly higher in shrimp suffered from WFS (Fig. 4c). The prediction accuracy reached 84.8%. These findings provided novel and direct evidence that the dysbiotic IM is a causal factor for WFS.

### Diet supplementary metabolites were relevant to IM dysbiosis

To validate the causal relationship between metabolites and IM dysbiosis, shrimp was challenged by nine types of WFS-enriched metabolites (caprylic acid, pentadecanoic acid, oleic acid, capric acid, lithocholic acid, linoleic acid, myristic acid, *N*-acetyl-d -glucosamine and succinate) in dietary supplementary for 1 week. Compared to the control group (Ctrl, without supplementary), the Shannon index significantly decreased under caprylic acid, myristic acid, and *N*-acetyl-d-glucosamine supplements (*P* < 0.05) (Additional file 2: Figure S9a). There was a clear separation in IM among Ctrl and metabolite-supplementary groups (Additional file 2: Figure S9b). Again, the disease-discriminatory genus *Vibrio* was overrepresented under pentadecanoic acid and oleic acid supplementary (*P* < 0.05), and *Candidatus* Bacilloplasma significantly increased with capric acid, lithocholic acid, succinate, and *N*-acetyl-d-glucosamine supplement (*P* < 0.05) (Additional file 2: Figure S10). Conversely, the control-enriched *Shewanella* decreased under caprylic acid, pentadecanoic acid, capric acid, and succinate challenges, and the proportion of *Chitinibacter* was significantly reduced under caprylic acid, pentadecanoic acid, oleic acid, and succinate supplementary (Additional file 2: Figure S10). The POD values were significantly higher in the metabolite-supplementary groups than in Ctrl (Additional file 2: Figure S11). Taken together, dietary supplementary of the tested metabolites exerts a detrimental effect on shrimp IM, which are consistent with the change pattern between healthy and WFS shrimp in the field. Thus, diet nutrition alteration is related to the development of IM dysbiosis.

### Molecular ecological networks of control and WFS

Molecular ecological network was constructed to understand species interactions of the IM after IMT (Fig. 4g). The WFS-related IM (WFS, C + W1, and C W2 groups) showed lower average connectivity and higher average path lengths than the control-related IM (control, C + P1, and C + P2 groups) (Additional file 2: Figure S12), indicating that species interactions of WFS were less cooperative and complex. In control-related networks, majority of the nodes belonged to *Proteobacteria* species, while the major nodes in WFS shifted into different phyla, including *Bacteroidetes* and *Chloroflexi*. The nodes with the greatest number of neighbors that could be considered as important hubs of the network belonged to *Vibrio* OTU1, *Shewanella* OTU3, *Candidatus* Bacilloplasma OTU4, and *Chitinibacter* OTU23 (Additional file 1: Table S9). These OTUs contained more distinctive neighbors in WFS-related groups, suggesting the interactions among the major species could be conserved to a certain extent but that most interactions could be swapped in control and WFS.

### Ecological processes governing the microbial community assembly in shrimp IM

The standardized effect size of the mean nearest taxon distance (ses.MNTD) measure was used to determine which processes govern the assembly of microbiota in shrimp intestine. Compared to the healthy shrimp, the deterministic process weakened in disease shrimp (Additional file 2: Figure S13). A null model analysis revealed that the relative contributions of homogeneous selection and heterogeneous selection processes were 11.3 ± 5.3% and 14.0 ± 5.2% in healthy shrimp, which were less pronounced in diseased shrimp (3.3 ± 3.0% and 6.5 ± 4.8%) (Additional file 2: Figure S14). After receiving IMT from WFS donors, the contribution of drift process was more than a half of the variation in C + W1 group and C + W2 group (64% and 75%). Meanwhile, homogenizing dispersal was the main ecological process that governed the IM in C + P1 (67%) and C + P2 (77%). Compared to C + P1 and C + P2 groups, homogenous selection and heterogeneous selection processes were less pronounced in shrimp suffered from WFS in C + W1 group (0% and 15%) and C + W2 group (0% and 7%). Additionally, the contribution of drift process was lower in W + C group (67%) as compared to W + P group (72%), while the relative contributions of homogenous selection and heterogeneous selection were higher in W + C group (20% and 7%). Collectively, shrimp WFS significantly counteracted (*P* < 0.05, paired *t* test) the relative importance of determinism (homogenous selection and heterogeneous selection) of IM (Additional file 1: Table S10).

## Discussion

The traditional Koch’s postulates are not valid to bring out a set of criteria for judging the cause of some complex non-infectious diseases. Here, we provide solid evidences that dysbiosis in IM causally leads to WFS, as comprehensively supported by compositional, functional, metabolomic aberrations, and IMT. This is the first attempt to validate the causal roles of dysbiotic IM in shrimp disease, which exemplifies the proposal of ‘microecological Koch’s postulates.’

To better understand the etiology of complex diseases, the Koch’s first postulate should expand the definition of pathogen from the presence of single organism in all cases to the presence of microbial dysbiosis in all individuals [20]. In other words, a dysbiotic microbiota should be found in similar composition in all diseased individuals. Some prior studies suggest that shrimp IM is strongly associated with developmental stage and environment [21, 22], yet the IM structure and assembly patterns tend to be similar during disease progression [15]. Consistently, decreased α-diversity and altered IM composition were detected in both fields (Fig. 1). It has been proposed that microbiota-targeted biomarkers serve as a powerful tool for disease diagnosis [21, 23]. The POD value successfully discriminated WFS from control by the optimal 167 OTUs markers (Fig. 1g), and further achieved accurate classification for distinguishing shrimps receiving IMT (Fig. 4c) and metabolite-supplements (Additional file 2: Figure S11) from control shrimp. Thus, WFS contribute a similar dysbiosis in the IM, irrespective the cause of disease. There are evidences that *Vibrio*, *Candidatus* Bacilloplasma, *Aeromonas*, and *Photobacterium* species are opportunistic pathogens for shrimp, which could cause gastrointestinal diseases [14, 24]. By contrast, *Chitinibacter* spp. have been applied as probiotics due to their capacity of chitin derivatives [25]. Consistent with these findings, increased abundances of the former opportunistic pathogens and decreased abundance of the later probiotics were the common features associated with WFS shrimp. The divergent changes in these populations might contribute the observation that a decline of pathways conferring lipoic acid metabolism and mineral absorption in WFS. This pattern was consistent with a previous study that lipid, carbohydrate, and amino acid metabolism were retarded in WFS shrimp [14]. In addition, the enrichment of bacterial chemotaxis and flagellar assembly pathways hint at pathogens growth and infectious diseases development [26], which were found in WFS shrimp (Additional file 2: Figure S4b). Indeed, the IM functional alterations were frequently linked to shrimp disease [27]. Meanwhile, some sorts of metabolites, especially lipids and organic acids, were found to be related to the IM alteration in WFS (Additional file 2: Figure S5). It is worth to emphasize that the dietary supplemented metabolites ultimately altered the microbial composition and diversity. Similarly, it has been proved that lipids exert an effect on the IM composition and diversity in shrimp [28]. Here, the integration of multiomics advanced the identification of key features in the IM and WFS. That is, WFS-associated dysbiosis in the IM led to the aberration of metabolites, which was validated by metabolite-supplements, resulting in similar IM and shrimp disease signs (Fig. 2c, Additional file 2: Figure S11). Collectively, these findings demonstrate common characterization, such as dysbiotic IM and its mediated functions, which were universal in WFS shrimp.

The dysbiotic IM in recipients can be retrieved from transplantation from WFS shrimp, thereby leading to similar symptom as in WFS shrimp. Here, shrimp that received the first and subsequent IMT from WFS donors finally exhibited similar clinical signs and histological pathology as these in WFS shrimp (Fig. 3), which evidenced that dysbiosis in IM contribute to WFS. Several studies have reported that the altered microbial composition is inconsistent among individuals and stages after FMT [20, 29]. Under this premise, it is necessary to recharacterize that the dysbiotic composition remains stable in the newly WFS shrimp. Notably, the features of lower α-diversity, increased abundance of opportunistic pathogens, and decreased proportion of probiotics (Fig. 4), as well as higher POD values (Fig. 4c), consistently recaptured in the WFS shrimp received the first and subsequent IMT. There is evidence that dysbiosis in the whole ecosystem (the microbiota, age, nutrition, etc.) leads to several complex diseases (e.g., obesity, IBD) [30, 31], explained by the ecological Koch’s postulates. Here, dysbiosis in the IM, including compositions, functional potentials, and metabolites, is a common feature in all WFS individuals. Intriguingly, these aberrations as well as similar disease symptoms were recharacterized by IMT. In consideration of the non-infection of WFS and the limited information about the shrimp genome, immunity, etc., an advisable interpretation, the microecological Koch’s postulates (a microbiota dysbiosis, a disease), is appropriate for the etiology of WFS.

To further investigate the community assembly patterns of the causal role of IM dysbiosis in WFS, molecular ecological networks and null community modeling were conducted. The clear stratification networks were observed between healthy and diseased shrimp, suggesting that shrimp disease substantially disturbed the balance of interspecies interaction [32]. By this logic, the smaller and less complex species interactions were indicative of a relatively unstable and less connective (Fig. 4g). It is known that stability and connectivity were related to the ecological assembly pattern [15], of which drift process increases when disease occurs [21]. Thus, an increased drift process could facilitate the overgrowth of opportunistic pathogens in WFS shrimp. The same pattern was found in shrimp suffered from WFS, showing that the contribution of drift process was higher in shrimp suffered from WFS (Additional file 2: Figure S14). By contrast, selection leads to convergent community compositions as a result of a consistent host filtering [33], whereas shrimp disease could weaken these selective pressures [21]. Consistent with this assertion, the relative contributions of homogenous and heterogeneous selection process were lower in WFS shrimp compared with corresponding healthy cohorts (Additional file 2: Figure S14). Accordingly, shrimp WFS reduces the filtering effects on the colonization of external species, thereby resulting in a less homogeneous IM community.

## Conclusion

**Fig. 5:**
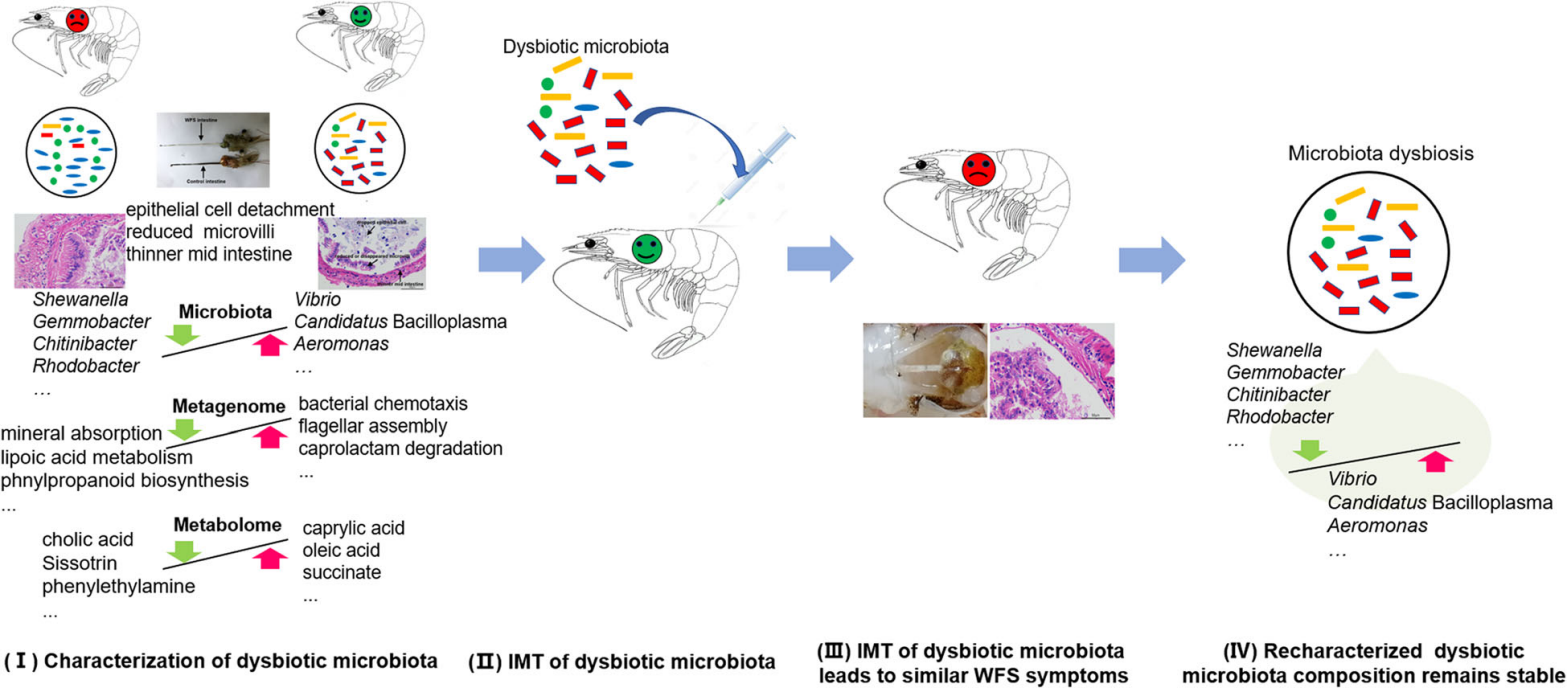
A graphic summary of the study design and the proposal of microecological Koch’s postulates in WFS. Firstly, the dysbiotic microbiota is characterized in similar composition in all diseased individuals. Then, the dysbiotic microbiota can be retrieved and transplanted to health individuals. The third postulate is that the transplanting IM can lead to similar symptom as in diseased ones. Lastly, it is necessary to recharacterize that the dysbiotic composition remains stable in the newly diseased individuals

In summary, we exemplify the causative role of IM dysbiosis in the occurrence of shrimp WFS. Thus, we synthesized our study design and main findings into a conceptual model as depicted in Fig. 5. The microecological Koch’s postulates for WFS shrimp are (I) the similar dysbiotic IM is characterized in all WFS shrimp; (II) the dysbiotic IM can be retrieved and transplanted to health shrimp; (III) the transplanting IM can lead to similar symptom as in WFS shrimp; and (IV) the recharacterization of the composition of the dysbiotic IM is consistent in the newly WFS shrimp. These principles are validated by multi-omics data, revealing the causality between IM dysbiosis and the occurrence of WFS, which open new cognition on the disease etiology from an ecological perspective in aquatic animals.

## Methods

### Study subjects and sample collection for IM analysis

Shrimp with an average length of 9 cm were collected from four earthen ponds in Zhuhai, Guangdong, China (22.38° N, 113.23° E). Each pond was approximately 2650 m^2^ and the depth was 1.0 m. The water temperature ranged from 28 to 31 °C. There was no antibiotic application during the culture period. Some probiotics, including *Lactobacillus* and *Bacillus*, have been mixed with feed and applied to ponds twice a week. Seventy-five healthy shrimp (control group) and 84 white feces syndrome shrimp (WFS group) were included in this study to compare the IM communities between the two statuses. To illustrate the relationship among microbiomes, metagenomes, and metabolomes, 20 shrimp’s intestines were put into a 5 mL tube containing 20 μL 1% (w/v) sodium azide solution and mixed thoroughly as one biological sample. Seven control samples and six WFS samples were collected for metagenomic and metabolomic analysis.

Shrimp surface was sterilized with 70% ethanol and their intestines were aseptically dissected as described elsewhere [34]. For histological examination, nearly 2 mm of shrimp intestine was cut off and soaked with Davidson’s fixative and processed for tissue sections of the intestine stained with hematoxylin and eosin (H & E) by light microscopy using standard methods [35]. For DNA extraction, the intestine was put into a 2 mL centrifuge tube which contained sterile glass beads and 1.5 mL PBS buffer. The tube was thoroughly vortexed for 3 min and centrifuged at 10,000 rpm for 1 min. Samples were immediately stored at − 80 °C before DNA extraction.

### IMT procedure in shrimp and sampling

IMT experiments were followed the methods developed in zebrafish and shrimp [36, 37]. Intestinal samples from healthy shrimp and WFS shrimp were selected as the control donors and WFS donors. Intestine sample was placed into a sterile tube containing 600 mL sterile PBS, and then was homogenized thoroughly. The mixture was centrifuged at 800 rpm for 1 min and the supernatant was transferred into a new centrifuge tube. The supernatant was also filtered through a 0.22 μm filter (Pall, USA) to collect the IM filtrate without bacteria.

Recipient shrimp were reared at a density of 30 shrimp in a 300 L tank. Shrimp received IM by IMT following previous reports [37, 38]. Using an automatic pipette, 5 μL of mixture was introduced in the shrimp anal cavity dispensing the IM mixture slowly to avoid traumatic effects. Starting 12 h after the IMT process, the shrimp were fed twice a day with a commercial pelleted feed.

Control shrimp were selected as recipients for four groups (*n* = 30 shrimp per group): IMT by using (I) PBS (C + P1); (II) IM from WFS donors (C + W1); (III) IM from control donors (C + C); (IV) IM filtrate without bacteria from WFS donors (C + WF). To reveal whether the dysbiotic IM composition was stable in shrimp suffered from WFS, six shrimp in C + W1 group were chosen to be WFS donors for the subsequent IMT. Shrimp were randomly distributed to two groups (*n* = 30 shrimp per group): IMT with (I) PBS (C + P2) and (II) IM from C + W1 WFS donors (C + W2). For further evidence of the relationship between IM and WFS, the WFS shrimp were received IMT from the health shrimp donors. The WFS shrimp were assigned to two groups (*n* = 22 shrimp per group): IMT with (I) PBS (W + P); (II) IM from control donors (W + C).

Recipient shrimp transferred with IM were kept feeding twice per day. Intestine histopathology and IM profiles of the recipients were investigated.

### Dietary supplement with altered metabolites to shrimp IM

To determine the effects of altered metabolites in regulating shrimp IM, shrimp were given feed supplemented with the WFS-enriched metabolites. For the feed supplementary experiment, we choose the metabolites which were not only enriched in WFS, but also correlated to the altered genera. Therefore, we finally chose nine metabolites for the feed supplementary experiment. Shrimp were reared in the pond and fed three times a day with a control commercial feed (Haida, China). After 3 days of adaptation to this feed, shrimp were fed with 2% supplement of (I) caprylic acid; (II) pentadecanoic acid; (III) oleic acid; (IV) capric acid; (V) lithocholic acid; (VI) linoleic acid; (VII) myristic acid; (VII) succinate; and (IX) *N*-acetyl-d-glucosamine. A group with no metabolite supplement was set as control (Ctrl). Shrimp were given fed with different supplementary for seven days. At the end of the experiment, shrimp intestines were aseptically dissected for IM analysis as described above.

### High throughput sequencing of 16S rRNA gene

Total DNA was extracted by the QIAamp PowerFecal DNA Kit (QIAGEN, USA) following the manufacturer’s directions. The concentration and purity of total DNA were determined by NanoVuePlus Spectrophotometer (GE Healthcare). The primer pair 515F (5′-GTGCCAGCMGCCGCGGTAA-3′) and 806R (5′-GGACTACHVGGGTW TCTAAT-3′) were used to amplify the V4 region of 16S rRNA gene, which was modified with a barcode tag containing a random 6-base oligos. Sequencing libraries were generated using TruSeq DNA PCR-Free Sample Preparation Kit (Illumina, USA) and the library quantity was determined by Qubit 2.0 Fluorometer (Thermo Scientific, USA). The libraries were sent for sequencing by a Hiseq2500 platform (Illumina, USA), which was conducted by Novogene Bioinformatics Technology Co., Ltd. (Beijing, China). Raw data generated from Hiseq2500 platform were paired-end reads.

To merge reads of the same DNA fragment, FLASH (Version 1.2.7) was used to gain the splicing sequences [39]. Based on the unique barcode, sequences were subjected to the removal of the barcode and primer sequence by QIIME (Version 1.7.0) [40]. To control the sequencing quality, raw tags with low quality (quality value ≤ 19, homopolymers ≥ 3 bases, and sequence length ≤ 200 bp) were filtered by QIIME. Tags were compared with Gold database (Version 7) [41] by UCHIME algorithm to remove chimera sequences and then the effective reads were finally gained.

Sequences with more than 97% similarity were assigned as the same OTU [42]. The GreenGene Database (Version gg_13_5) was used as a reference database to align the sequences and later the taxonomic information was annotated by RDP classifier (Version 2.2) with 80% confidence [43]. Alpha diversity, including Shannon, Simpson, and Chao1 index, was calculated with QIIME and displayed with R software (Version 2.3.0). Bray-Curtis distance was used to evaluate the species complexity differences of samples.

### Metagenomic sequencing and gene catalog construction

Sequencing libraries were generated using NEBNext Ultra DNA Library Prep Kit for Illumina (NEB, USA) and index codes were added to attribute sequences to each sample. The DNA sample was fragmented by sonication to a size of 300 bp, then DNA fragments were end-polished and ligated with the full-length adaptor for Illumina sequencing with further PCR amplification. The libraries were analyzed for size distribution by Agilent2100 Bioanalyzer (Agilent, USA), and then were sequenced by Illumina Hiseq2500 platform in MAGIGene Co., Ltd. (Shenzhen, China).

Quality control was conducted by Trimmomatic (Version 0.38) [44]. The reads aligned to the NCBI non-redundant (NR) database were removed with MEGAHIT (Version 1.05) [45]. The remaining high-quality reads were used for further analysis. The assembly of reads was executed using MEGAHIT de novo. For each sample, a series of k-mer values (49 to 87) were used and the optimal one with the longest N50 value were chosen for the remaining scaffolds. The clean data were mapped against scaffolds using MEGAHIT. Unused reads from each sample were assembled using the same parameters. Genes (minimum length of 100 nucleotides) were predicted on scaftigs longer than 500 bp using Prodigal (Version 2.6.3) [46]. Then, a non-redundant gene catalog was constructed with Linclust (Version 2.0) using a sequence identity cut-off of 0.9. To determine the abundance of genes, reads were realigned to the gene catalog with BBMap (Version 37.68, https://sourceforge.net/projects/bbmap). Only genes with ≥ 2 mapped reads were deemed to be present in one sample. The abundance of genes was calculated by counting the number of reads and normalizing by gene length.

All genes were aligned to the KEGG database using DIAMOND (Version 0.7.9.58) with the *E* value of 1E-4 [47]. The gene was then assigned to the KEGG orthology by the highest scoring annotated hits. The abundance of KEGG pathway was calculated by summing the abundance of genes annotated to the same feature.

### Metabolome analysis based on LC/MS of control and WFS

Shrimp intestines weight 100 mg were diluted with 1 mL mixture of methanol-acetonitrile-water (2:2:1, v/v). The mixtures were centrifuged at 13,000 rpm for 15 min at 4 °C, of which 100 μL of the supernatant was harvested for LC/MS analysis. The metabolic profiles were performed on an Agilent 1290 Infinity LC system (Agilent, USA). For chromatographic separation, C18 (2.1 mm × 100 mm × 1.9 μm) reversed-phase column (Thermo Scientific, USA) preheated at 25 °C was used. A prepared sample of 2 μL was injected and maintained at 25 °C for analysis. The gradient conditions for elution were at 95% acetonitrile for 0.5 min, 95–65% from 0.5 to 7 min of linear gradient, 65–40% from 7 to 8 min, 40% from 8 to 9 min, 40–95% from 9 to 9.1 min, and 95% from 9.1 to 12 min. The mobile phase for positive ion mode (ES+) and negative ion mode (ES−) was composed of water with 0.1% formic acid as solvent A, and acetonitrile with 0.1% formic acid as solvent B, and the flow rate was at 300 μL min^−1^. For mass spectrometric assay, Triple-TOF 5600 mass spectrometer (AB SCIEX, USA) was used to analyze the metabolite ions. The spray voltage was set to ± 5.5 kV and the ion source temperature was 600 °C. Masses ranging from 50 to 1000 ion mass (m/z) were acquired, and the resolving power was set to 60,000. The raw data of LC/MS was converted into m/z format and analyzed for non-linear retention time (RT) alignment, peak detection, and filtration. Maximal spectrum of continuous wavelet transform was used to correct baseline and detect peak positions. Impurity peaks and duplicate identifications were eliminated.

Compounds significantly different between groups were obtained at a variable influence on projection (VIP) > 1.5, and *P* value of *t* test statistics < 0.05 based on the peak intensities. The m/z values of these compounds were used to identify the metabolites corresponding to the featured peak in the Metlin metabolite database [48].

### Molecular ecological network construction between control and WFS

The correlation network was constructed from OTUs based on the Random matrix theory (RMT) [49, 50]. RMT-based approach is a powerful way to construct molecular ecology network in which represent various biological interactions [50]. By determining the most interacted microbial taxa, networks can also identify the keystone species that exert disproportional effects on microbial community structure and potential functions [51]. Based on within-module and among-module connectivity, topological roles of different nodes were divided into four categories, including (I) network hubs: nodes with Zi > 2.5 and Pi > 0.6; (II) module hubs: nodes with Zi > 2.5 and Pi ≤ 0.6; (III) connectors: nodes with Zi ≤ 2.5 and Pi > 0.6; and (IV) peripheral nodes: nodes with Zi ≤ 2.5 and Pi ≤ 0.665 [52], in which Zi and Pi respectively indicate how well a node connects to nodes within the same and other modules. The global network properties and individual node’s centrality was then displayed by Cytoscape (Version 3.3.0).

### Estimation of ecological processes

The mean nearest taxon distance (MNTD) was calculated to determine which processes govern the assembly of shrimp IM [53]. The obtained standardized effect size measure (ses.MNTD), which is also known as the negative nearest taxon index (NTI) to determine ecological processes that govern a community in terms of phylogenetic structures [54, 55], was calculated with Picante package in R. The mean distance between each taxon and its nearest neighbor (β-MNTD) was computed by random shuffling of OTUs and their abundances across phylogenetic tips, reflecting the dissimilarity between communities [53, 56]. Difference between the observed β-MNTD and the mean of the null distribution is referred as β-NTI. The β-NTI in combination with Bray–Curtis-based Raup–Crick (RC_Bray_) was further used to quantify the relative contributions of major ecological processes that determine the assembly of IM [53]. The relative influence of community turnover that was determined by homogeneous and heterogeneous selection was denoted by β-NTI < − 2 and β-NTI > + 2 fractions, respectively [57, 58]. The β-NTI and RC_Bray_ were used to estimate the contribution of homogenizing dispersal and dispersal limitation. If |β-NTI| < 2 but RC_bray_ > + 0.95 or < − 0.95, community turnover was governed by dispersal limitation or homogenizing dispersal processes [53]; the fraction of pairwise comparisons with |β-NTI| < 2 and |RC_Bray_| < 0.95 represented the component of compositional turnover was governed by undominated [59].

### Statistics analysis

To compare the difference between groups, statistical analysis was conducted by SPSS (Version 21.0). If data were normally distributed, a two-tailed Student’s *t* test was used to determine significance in experiments with only two groups, and analysis of variance (ANOVA) with Tuckey test was used to determine significant differences between multiple test groups. In cases where data were not normally distributed, a one-way ANOVA with Kruskal-Wallis test was performed. MRPP, ANOSIM, and PerMANOVA were conducted to statistically test whether there is a significant difference between two groups with vegan package in R [60, 61]. Permutational analysis of multivariate dispersions (PERMDISP) was for the analysis of multivariate homogeneity of group dispersions by vegan package in R [61]. Random forests regression was used to regress relative abundances of taxa in the temporal profiles of Control and WFS groups, using the following parameters with randomForest package in R (cv. fold = 10, step = 0.99, replication = 55, ntree = 5000). The POD value was defined as the ratio between the number of decision trees that was voted as ‘WFS’ and the number of total sampling trees (*n*_votes_/*n*_trees_). Spearman’s rank correlation was conducted to measure the correlation of two variables in SPSS. Beta-diversity comparison of metagenomic and metabolome was accomplished by using Procrustes transformations with PCoA based on Bray-Curtis distance with vegan package in R.

## Supplementary information

**Additional file 1.** Supplementary Tables S1-S10.

**Additional file 2.** Supplementary Figures S1-S14.

## Data Availability

The 16S rRNA gene sequencing data and metagenomic data used in this study are available in the NCBI Short Read Archive (https://www.ncbi.nlm.nih.gov/sra) under Bioproject PRJNA542015.

## Abbreviations

AHPND: Acute hepatopancreatic necrosis disease
ANOSIM: Analysis of similarities
EMS: Early mortality syndrome
FMT: Fecal microbiota transplantation
HPNS: Hepatopancreas necrosis syndrome
IBD: Inflammatory bowel disease
IM: Intestinal microbiota
IMT: Intestinal microbiota transplantation
KEGG: Kyoto encyclopedia of genes and genomes database
LC/MS: Liquid chromatography-mass spectrometry
MEN: Molecular ecological network
MRPP: Multiple-response permutation procedure
OPLS-DA: Orthogonal partial least-squares discriminant analysis
OTU: Operational taxonomic units
PBS: Phosphate buffer saline
PCA: Principal component analysis
PCoA: Principal coordinate analysis
PerMANOVA: Permutational multivariate analysis of variance
PERMDISP: Permutational analysis of multivariate dispersions
POD: Probability of disease
RMT: Random matrix theory
RT: Retention time
SIMPER: Similarity percentage analysis
VIP: Variable influence on projection
WFS: White feces syndrome
