# Supplementary material for: Microecological Koch’s postulates reveal that intestinal microbiota dysbiosis contributes to shrimp white feces syndrome
Source: Microbiome. 2020 Mar 10;8:32. doi: 10.1186/s40168-020-00802-3 (PMC7065354; doi:10.1186/s40168-020-00802-3)
Supplement: Supplementary file 3 — Additional file 2. Supplementary Figures S1-S14. [file 40168_2020_802_MOESM2_ESM.docx]

Supplementary Data for

**Microecological Koch’s postulates reveal that intestinal microbiota dysbiosis contributes to shrimp white feces syndrome**

Zhijian Huang, Shenzheng Zeng, Jinbo Xiong, Dongwei Hou, Renjun Zhou, Chengguang Xing, Dongdong Wei, Xisha Deng, Lingfei Yu, Hao Wang, Zhixuan Deng, Shaoping Weng, Satapornvanit Kriengkrai, Daliang Ning, Jizhong Zhou, Jianguo He

Correspondence to: Dr. Zhijian Huang, lsshzhj@mail.sysu.edu.cn;

Dr. Jianguo He, lsshjg@mail.sysu.edu.cn

**This supplementary data file contains the following:**

Supplementary Figures 1 to 14

**SUPPLEMENTARY FIGURES**


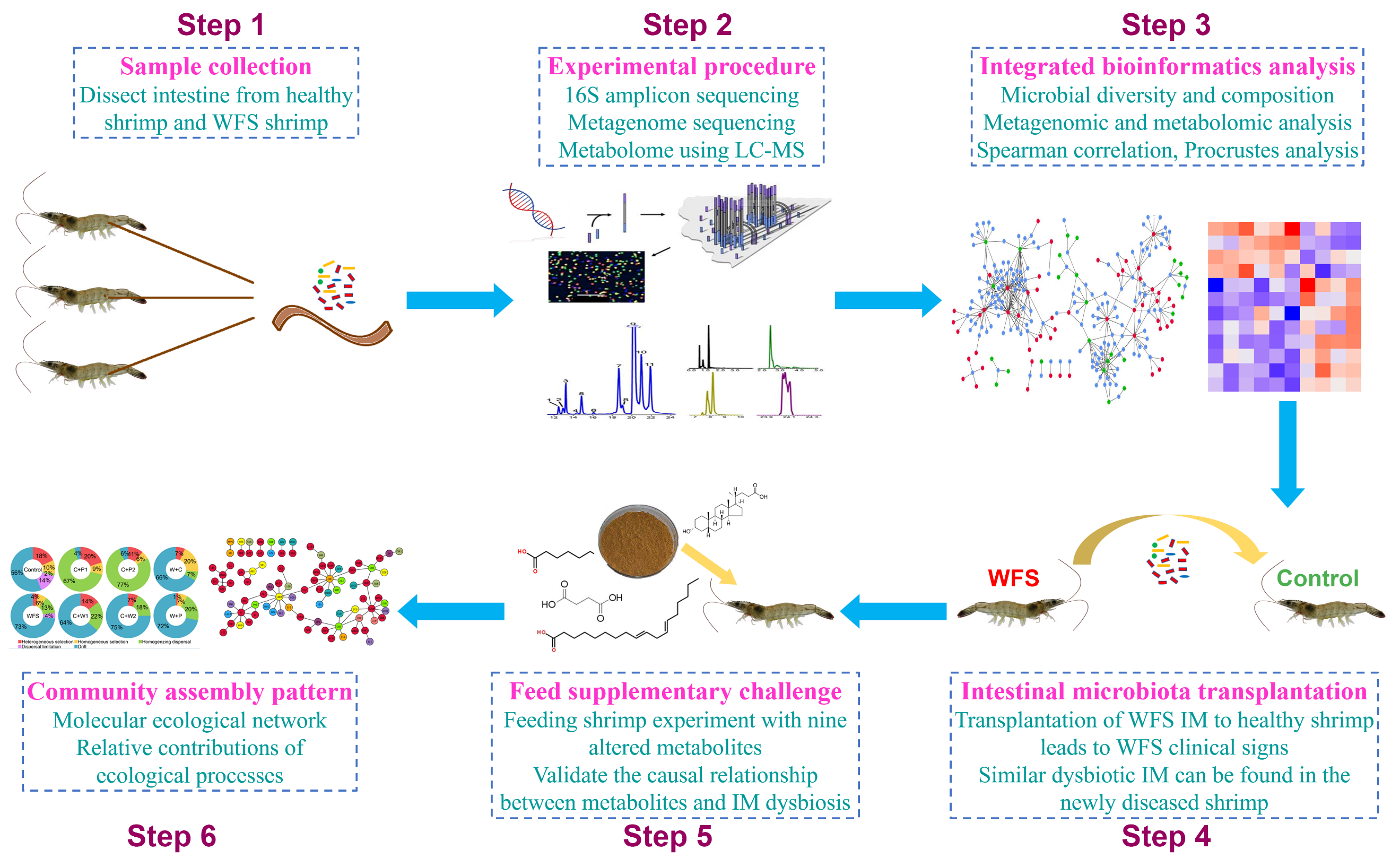


**Figure S1 Workflow for the comprehensive exploration of IM dysbiosis in WFS shrimp**

The working steps were numbered from Step 1 to Step 6. Step 1: Healthy shrimp (Control group) and white feces syndrome shrimp (WFS group) were collected, and their intestines were aseptically dissected. Step 2: To characterize the WFS features, comprehensive multi-omics experiments were conducted, including 16S amplicon sequencing, metagenome sequencing and metabolome using LC-MS. Step 3: With integrated bioinformatics analysis, dysbiotic IM was characterized in WFS shrimp. Step 4: To further demonstrate whether IM dysbiosis is a causal factor in WFS occurrence, IM from WFS donors were transplanted to healthy shrimp, which led the recipient shrimp to develop WFS signs. Moreover, the recharacterization of the composition of the dysbiotic IM is consistent in the newly WFS shrimp. Step 5: For validation of the causal relationship between metabolites and IM dysbiosis, shrimp was challenged by nine WFS-enriched metabolites. Step 6: Molecular ecological networks and null community modeling were conducted to further investigate the community assembly patterns of the causal role of IM dysbiosis in WFS.


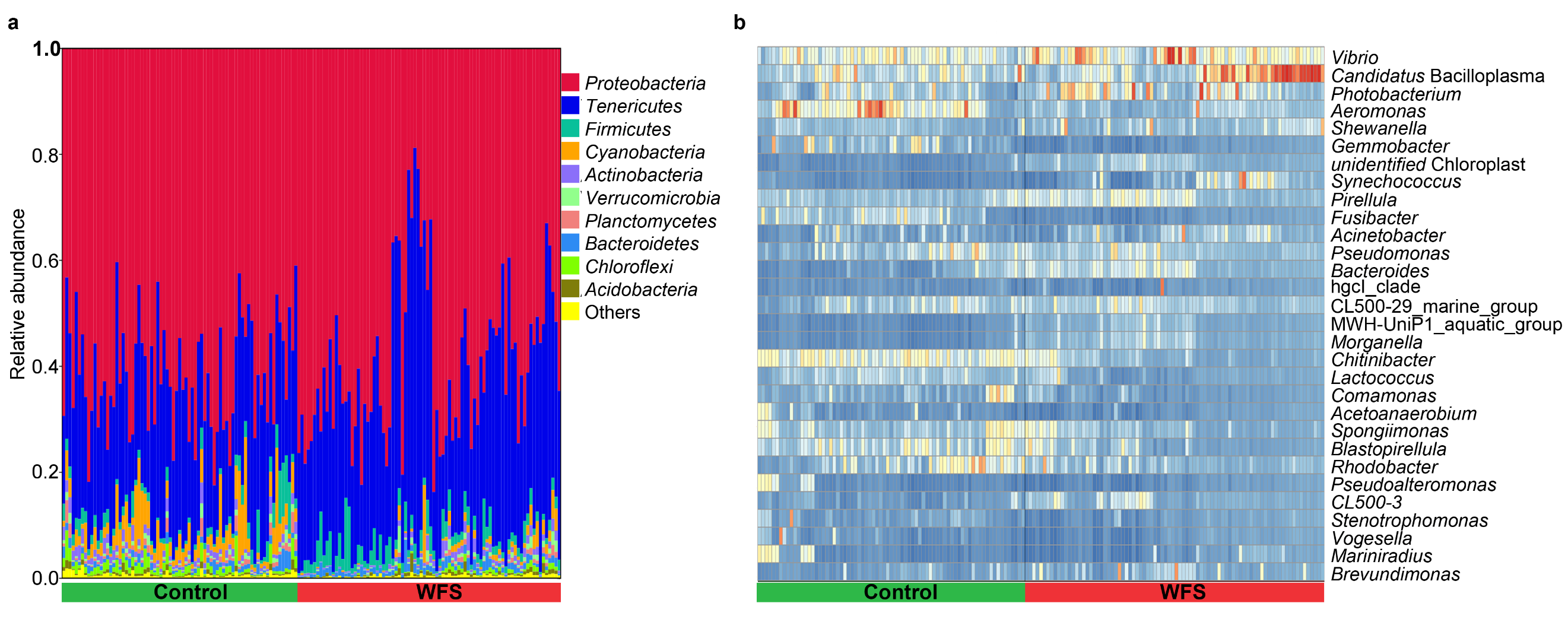


**Figure S2 Microbial composition of Control and WFS shrimp**

**a** The relative abundances of bacterial communities in Control IM and WFS IM at phylum level. **b** Heatmap of bacterial distributions in Control IM and WFS IM at genus level. Rows represent the 35 most abundant bacterial genera and the square-root-transformed relative percentage of each genus is depicted by color intensity. The relative abundance of each column was normalized to *Z* score in heatmap.


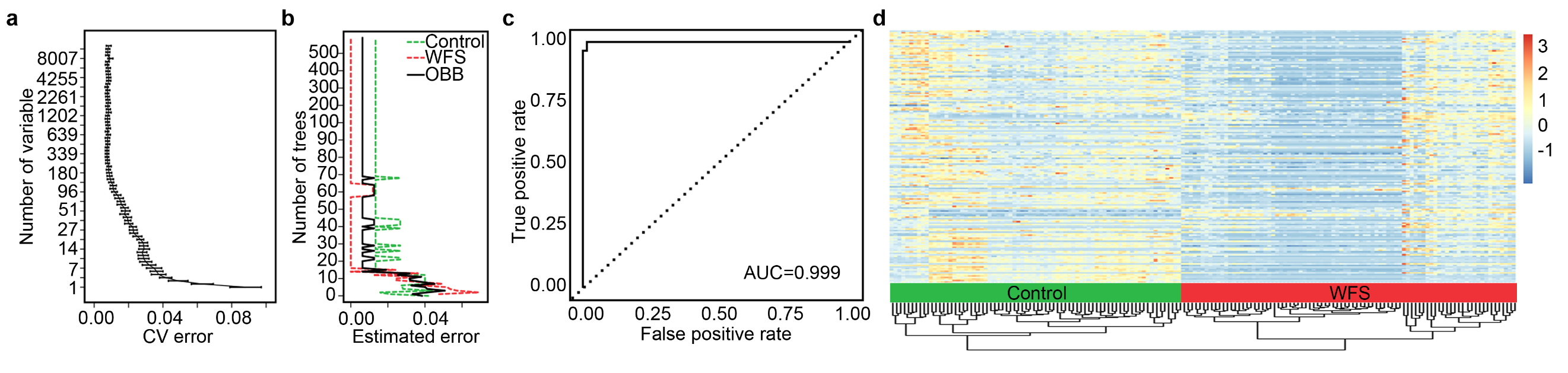


**Figure S3 Prediction of probability of disease for WFS by random forest models based on the OTUs markers.**

To detect unique OTUs markers for WFS, we conducted a 10-fold cross-validation on a random forest model between 75 Control samples and 84 WFS samples. **a** The 167 OTUs markers were selected as the optimal marker set by the CV-error curve. **b** The number for trees used for random forest model was assessed, which showed that the number larger than 500 was suitable for the model. **c** The area under curve (AUC) shows the classification of Control versus WFS as the numbers of variables increase. The classification is efficient as indicated by a high AUC value of 0.999. **d** Heatmap based on the 167 markers revealed that the WFS group was clearly distinct to the Control group.

**
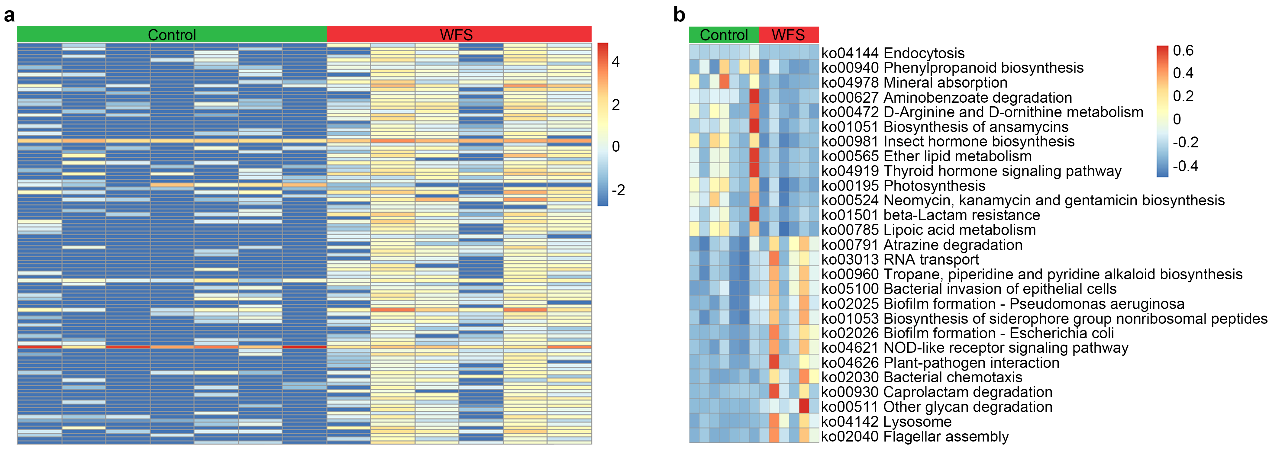
**

**Figure S4 Comparative analysis of microbial gene functions annotation between Control (n = 7) and WFS (n = 6).**

**a** The average abundance of the 111 KEGG orthologs differentially enriched in Control group and WFS group. **b** The average abundance of the 27 KEGG pathways differed significantly between Control group and WFS group.


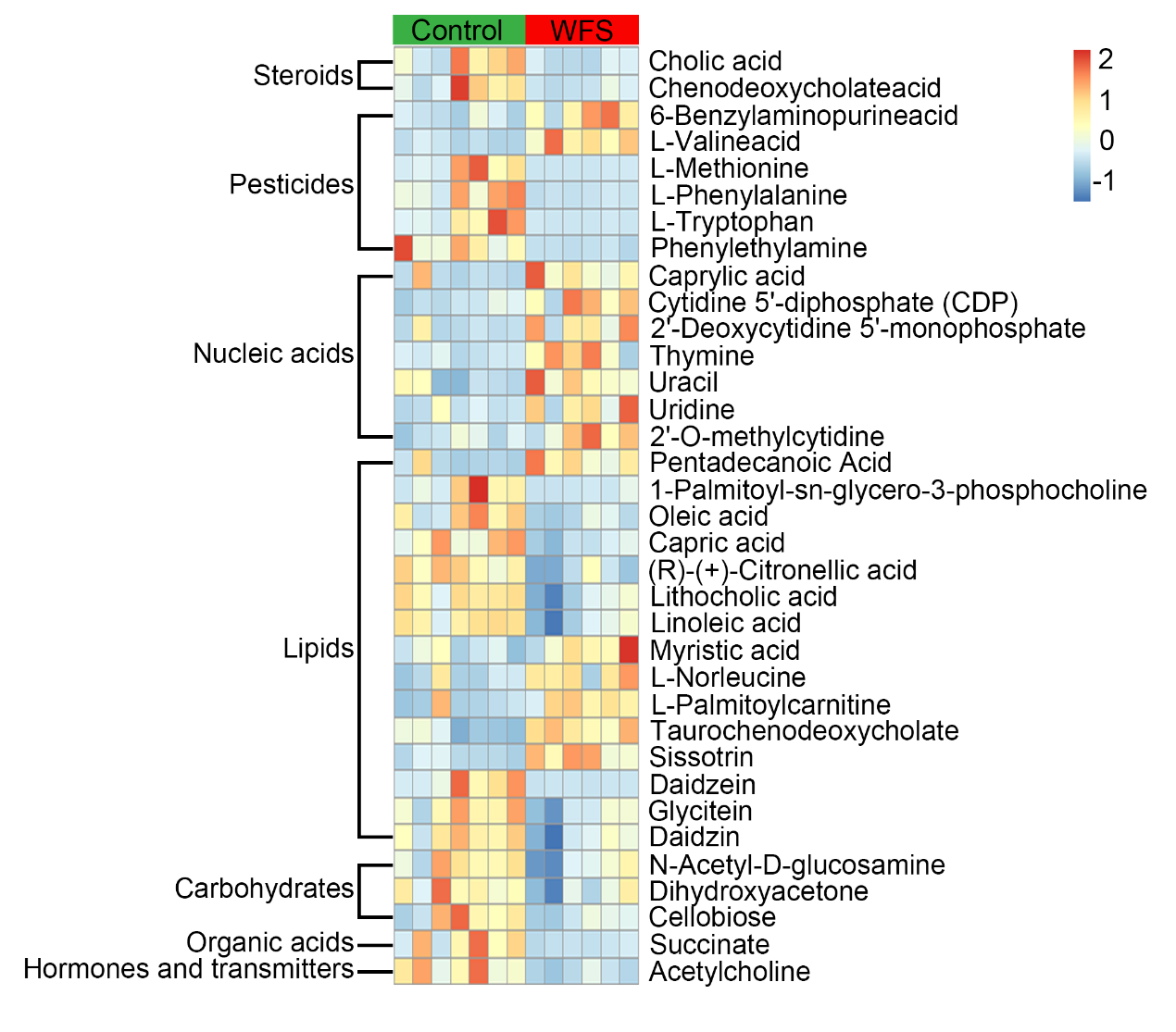


**Figure S5 Altered metabolites listed in KEGG database**

A total of 35 KEGG listed metabolites significantly altered in Control group and WFS group, including 20 metabolites were enriched in Control group, whereas 15 metabolites were enriched in WFS group.


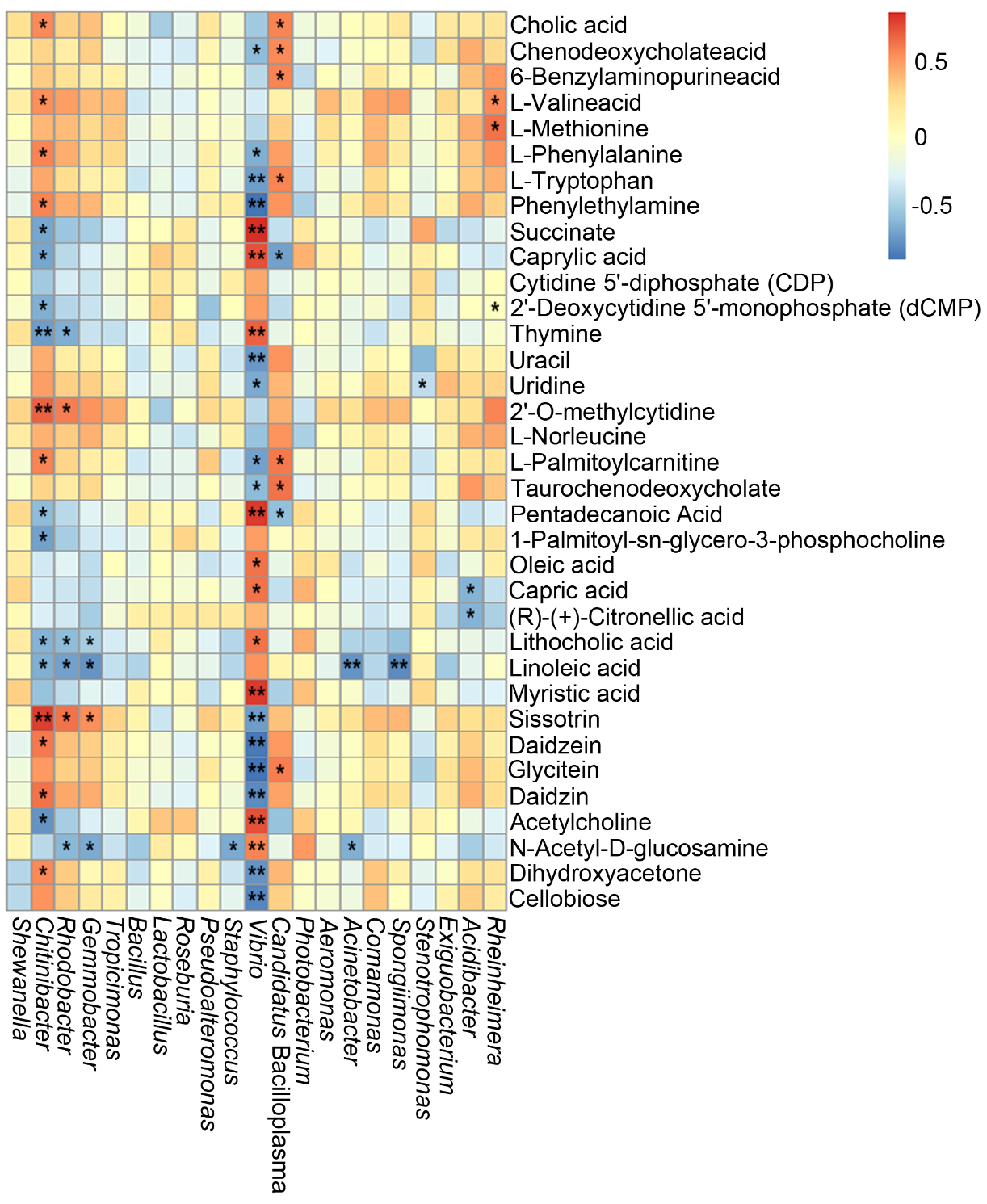


**Figure S6 The relationship of distinguished metabolites and genera.**

A total of 35 KEGG listed metabolites and 21 genera were differed from Control group and WFS group. The relationship among altered genera and metabolites is estimated by Spearman’s correlation analysis. Significant differences are indicated by asterisks (*, *P* < 0.05; **, *P* < 0.01).


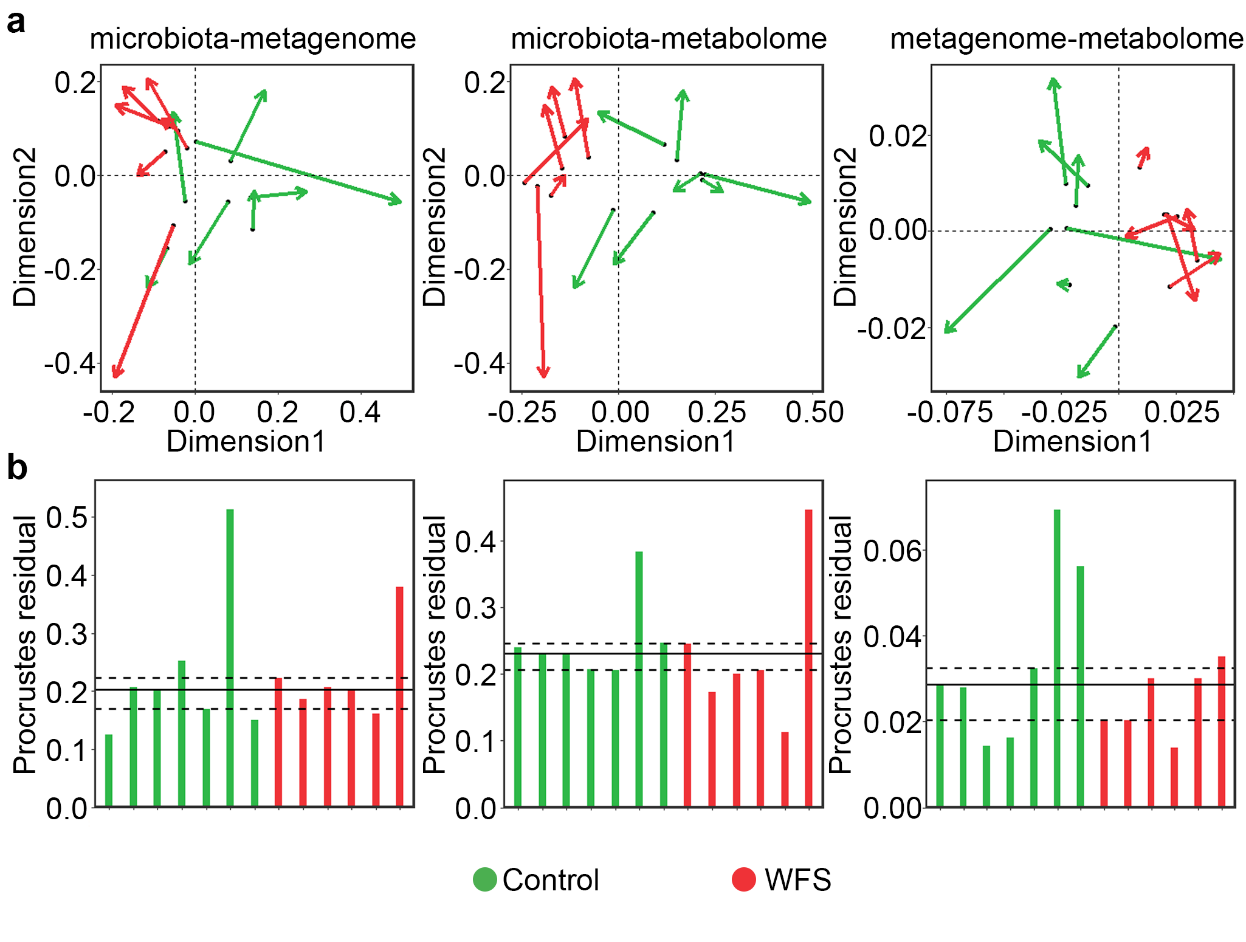


**Figure S7 The relationship among microbiota, metagenome and metabolome**

**a** The Procrustes correlation is based on the PCoA ordination of the microbiota, metagenome and metabolome matrices to calculate the association between two data matrices. **b** Bar chat of Procrustes residual. The residual value indicated the correlation value between the two data sets.


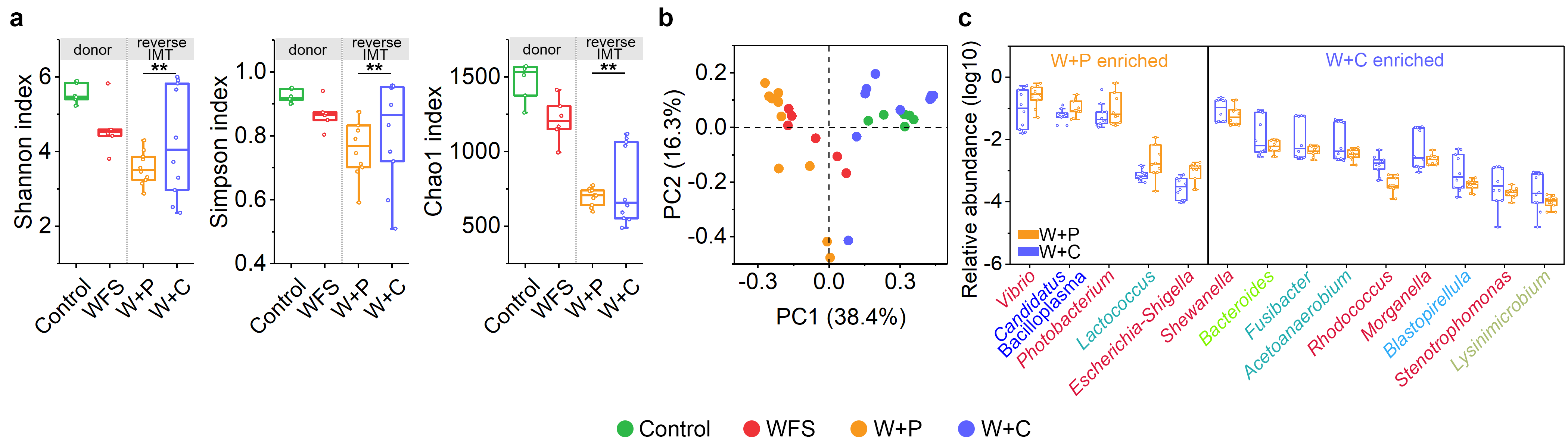


**Figure S8 Comparative analysis of the reverse IMT.**

**a** Recipient Control shrimp significantly increased with higher α-diversity (*P* < 0.05, Student’s *t*-test), Significant differences are indicated by asterisks (**, *P* < 0.01). **b** Control donors and recipient shrimps were clustered closely, separating from the WFS and PBS groups. **c** Boxplot comparing the abundance of altered genera after receiving the reverse IMT.


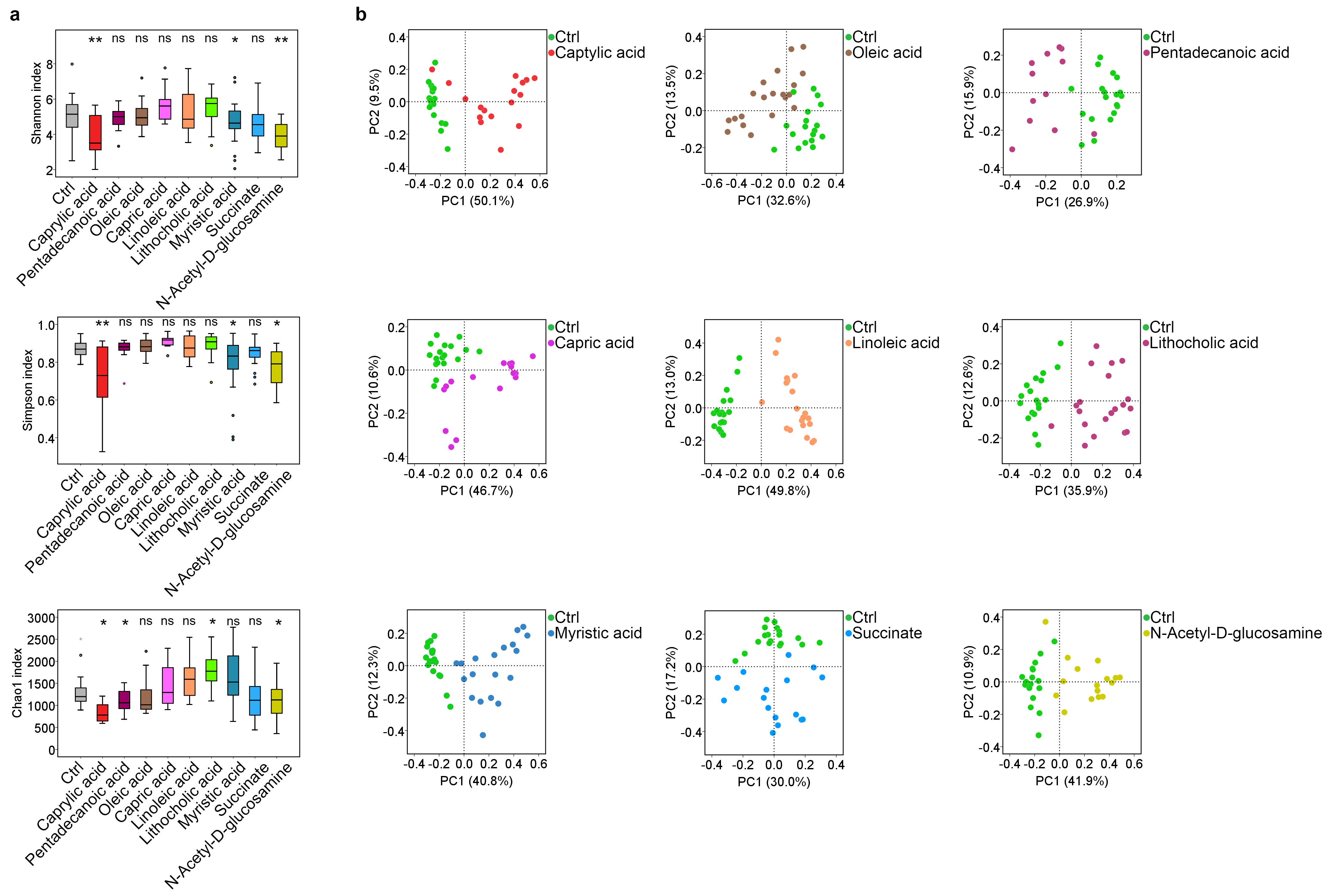


**Figure S9 Diet supplementary metabolites contribute to IM dysbiosis.** Shrimp was challenged by nine kinds of WFS-enriched metabolites (caprylic acid, pentadecanoic acid, oleic acid, capric acid, lithocholic acid, linoleic acid, myristic acid, N-acetyl-D-glucosamine and succinate). A group of shrimps without any metabolites supplement was set as control group (Ctrl). **a** The *α*-diversity decreased with caprylic acid and myristic acid supplement. **b** Nine metabolites that were enriched in WFS groups were supplemented to feed for one week. PCoA plot revealed that the microbial structure shifted after one week with supplemented metabolites. Asterisk denotes significant differences based on ANOVA.


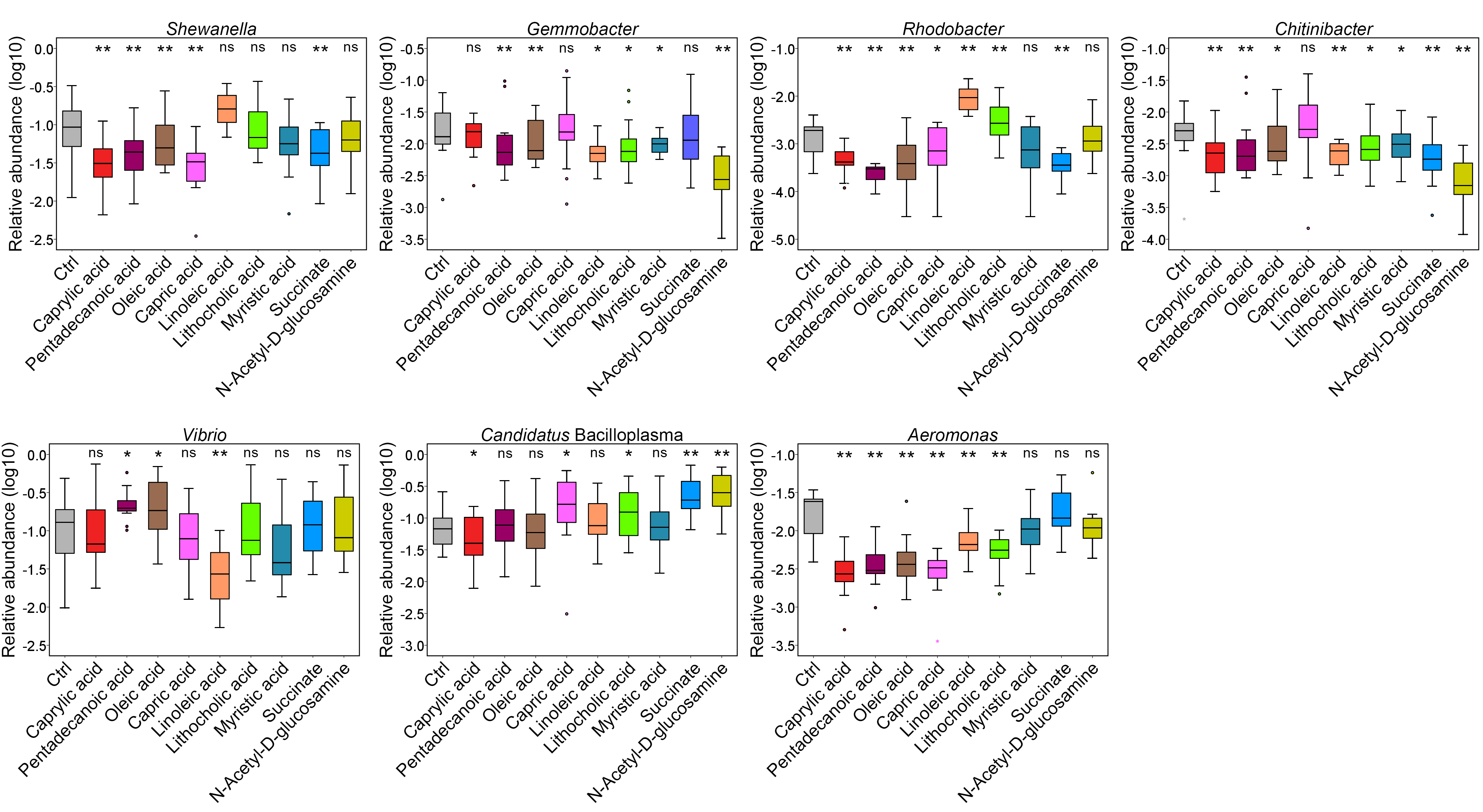


**Figure S10 Microbial composition alteration with supplemented metabolites**

Compared with the Ctrl group, the distinguished genera for WFS were altered with supplementary metabolites. The WFS-enriched genera (*Vibrio* and *Candidatus* Bacilloplasma) significantly increased in 5 metabolites, whereas the Control-enriched genera (*Shewanella*, *Chitinibacter*, *Gemmobacter* and *Rhodobacter*) significantly decreased.

**
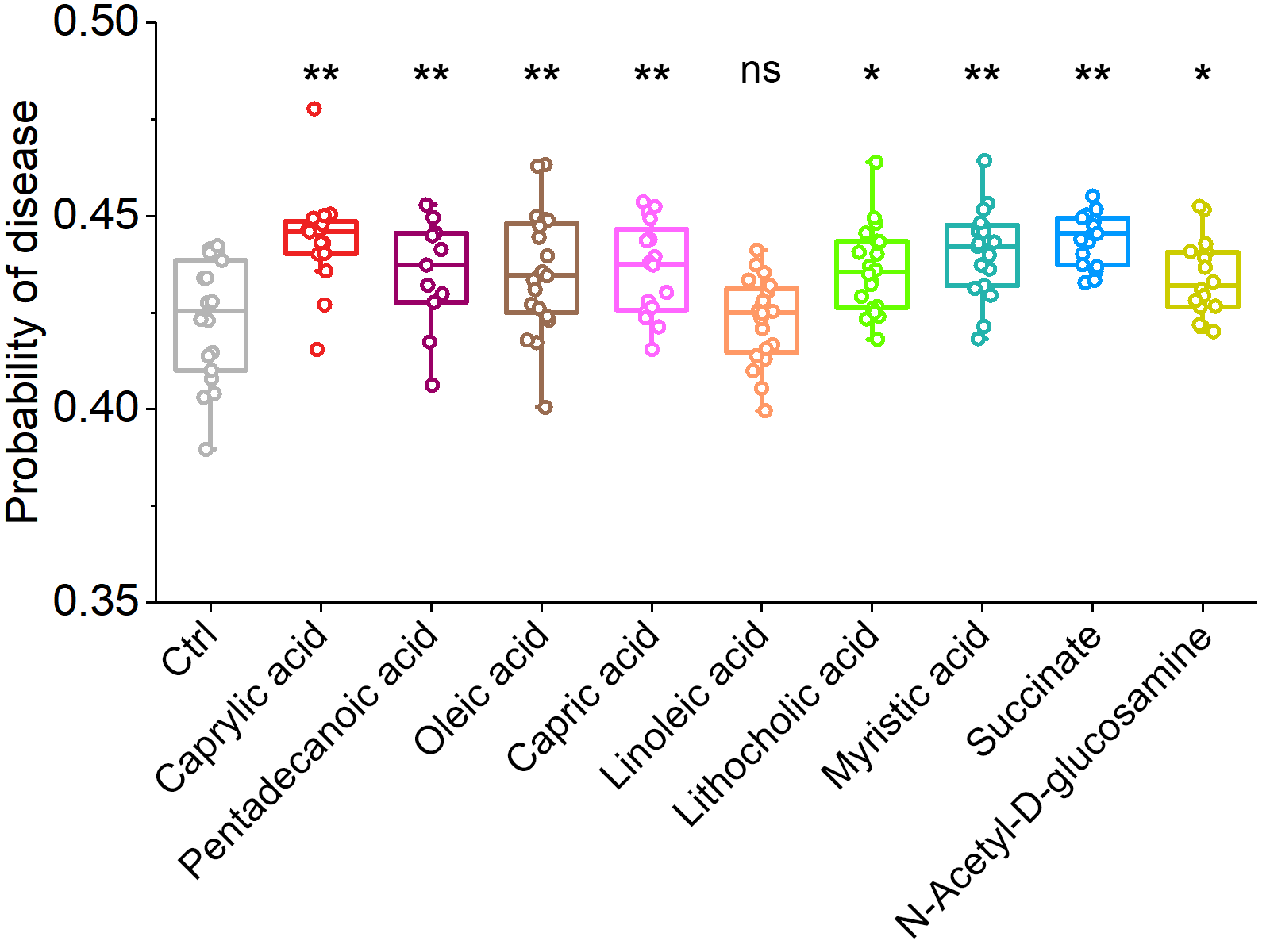
**

**Figure S11 Probability of disease was significantly higher after feeding with supplementary metabolites.**

Compared with the Ctrl group, the POD values of the metabolite groups were significantly higher than that of the Ctrl group. Significant differences are indicated by asterisks (*, *P* < 0.05; **, *P* < 0.01). No significant difference is indicated by ns.

**
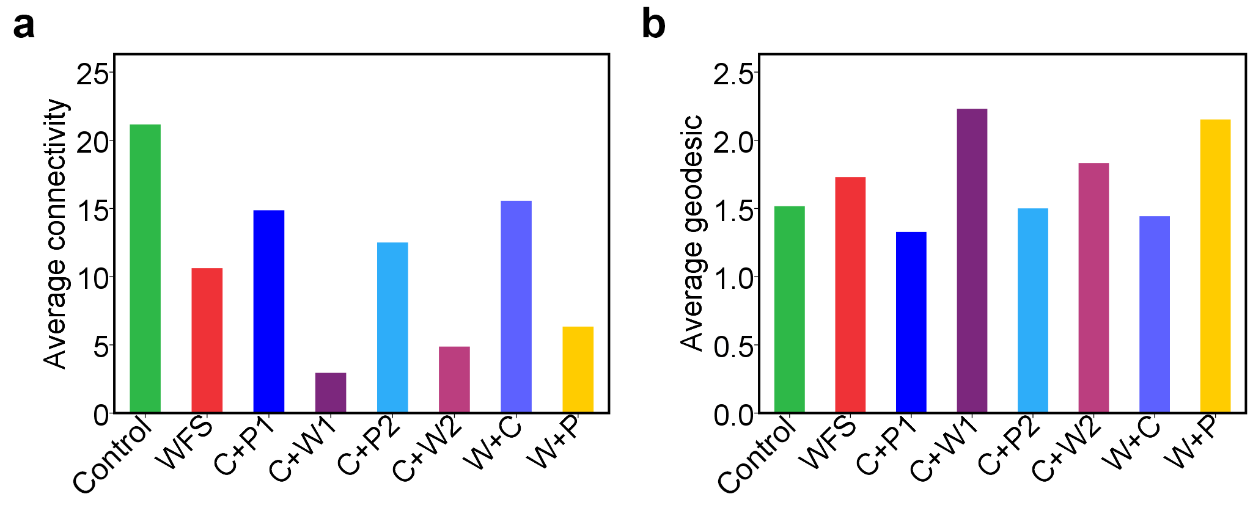
**

**Figure S12 Comparations of species interaction in different groups.**

The indexes describe the network topology in different groups. **A** The higher average connectivity value means the more complex network. The average connectivity in the Control-related IM (Control, C+P1, C+P2 and W+C groups) were higher than the WFS-related IM (WFS, C+W1, C+W2 and W+P groups). **B** The smaller geodesic value means all the nodes in the network are closer. The average geodesic in the Control-related IM were significantly lower than the WFS-related IM.

**
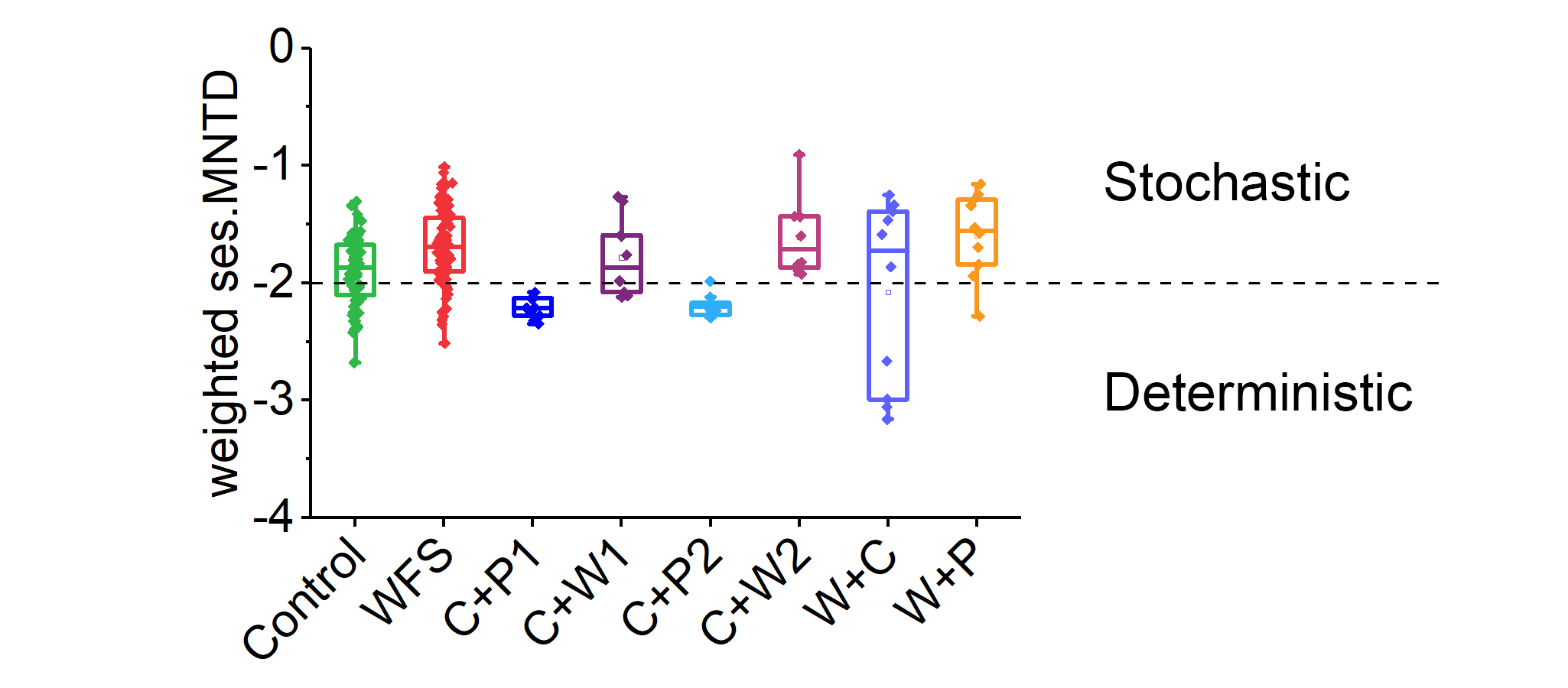
**

**Figure S13 Comparison of the contribution of the ecological processes.**

The processes govern the assembly of microbiota reflected by the weighted standardized effect size of the mean nearest taxon distance (ses.MNTD) value. The |ses.MNTD| > 2 are considered that determinism dominantly governs the community assembly, while |ses.MNTD| < 2 suggest that stochasticity is dominant.

**
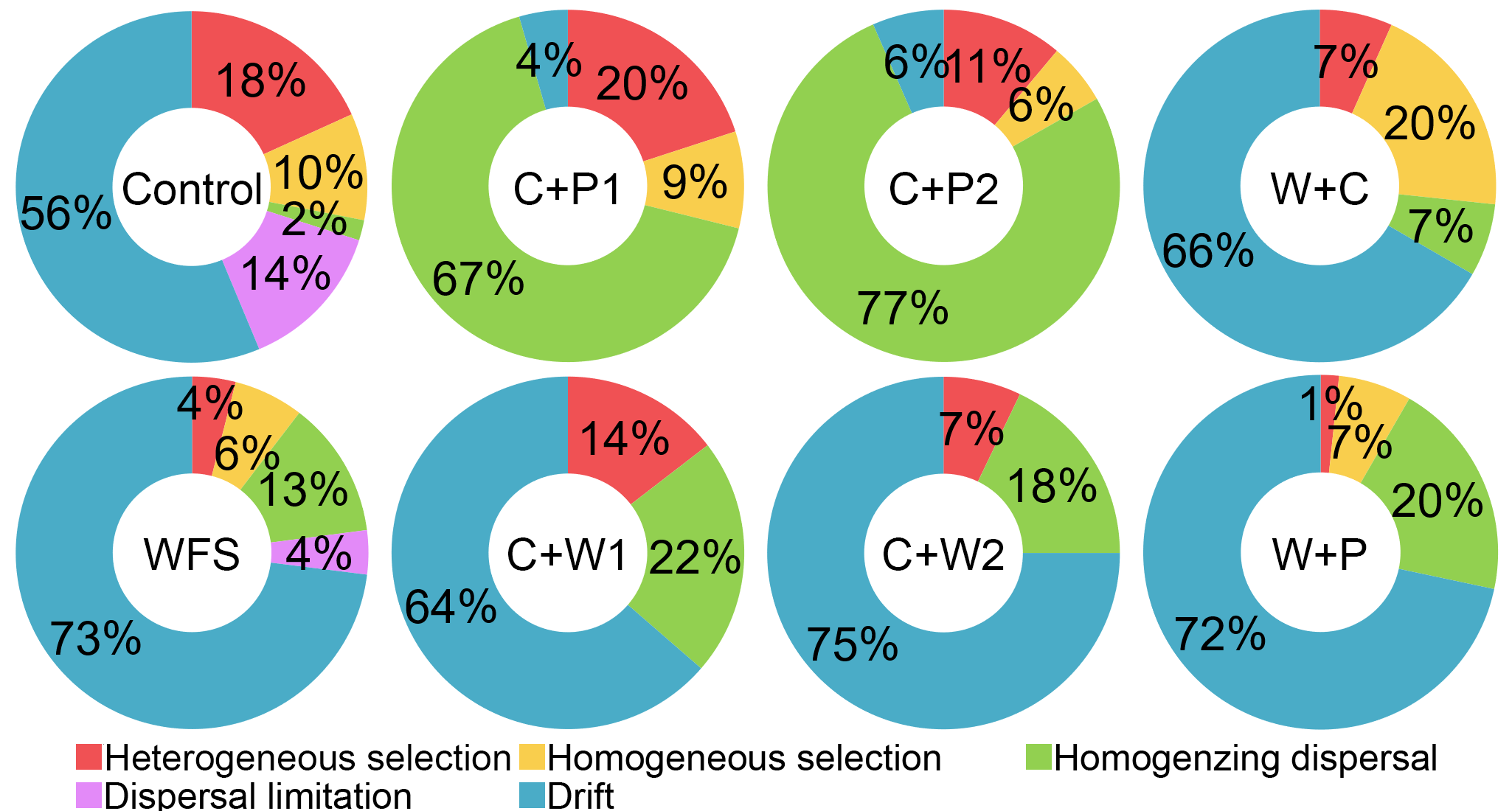
**

**Figure S14 Comparison of the contribution of the ecological processes.**

The ecological processes determine community assembly of intestinal microbiota in different groups. The homogenous selection process and heterogeneous selection process were less pronounced in WFS community assembly
